# Lysine provisioning by horizontally acquired genes promotes mutual dependence between whitefly and two intracellular symbionts

**DOI:** 10.1371/journal.ppat.1010120

**Published:** 2021-11-29

**Authors:** Xi-Yu Bao, Jin-Yang Yan, Ya-Lin Yao, Yan-Bin Wang, Paul Visendi, Susan Seal, Jun-Bo Luan

**Affiliations:** 1 Liaoning Key Laboratory of Economic and Applied Entomology, College of Plant Protection, Shenyang Agricultural University, Shenyang, China; 2 Centre for Agriculture and the Bioeconomy, Institute for Future Environments, Queensland University of Technology, Brisbane, Queensland, Australia; 3 Agriculture, Health and Environment Department, Natural Resources Institute, University of Greenwich, Central Avenue, Chatham Maritime, Kent, United Kingdom; University of Cambridge, UNITED KINGDOM

## Abstract

Horizontal gene transfer is widespread in insects bearing intracellular symbionts. Horizontally transferred genes (HTGs) are presumably involved in amino acid synthesis in sternorrhynchan insects. However, their role in insect-symbiont interactions remains largely unknown. We found symbionts *Portiera*, *Hamiltonella* and *Rickettsia* possess most genes involved in lysine synthesis in the whitefly *Bemisia tabaci* MEAM1 although their genomes are reduced. *Hamiltonella* maintains a nearly complete lysine synthesis pathway. In contrast, *Portiera* and *Rickettsia* require the complementation of whitefly HTGs for lysine synthesis and have *lysE*, encoding a lysine exporter. Furthermore, each horizontally transferred lysine gene of ten *B*. *tabaci* cryptic species shares an evolutionary origin. We demonstrated that *Hamiltonella* did not alter the titers of *Portiera* and *Rickettsia* or lysine gene expression of *Portiera*, *Rickettsia* and whiteflies. *Hamiltonella* also did not impact on lysine levels or protein localization in bacteriocytes harboring *Portiera* and ovaries infected with *Rickettsia*. Complementation with whitefly lysine synthesis HTGs rescued *E*. *coli* lysine gene knockout mutants. Silencing whitefly *lysA* in whiteflies harboring *Hamiltonella* reduced lysine levels, adult fecundity and titers of *Portiera* and *Rickettsia* without influencing the expression of *Hamiltonella lysA*. Furthermore, silencing whitefly *lysA* in whiteflies lacking *Hamiltonella* reduced lysine levels, adult fecundity and titers of *Portiera* and *Rickettsia* in ovarioles. Therefore, we, for the first time, demonstrated an essential amino acid lysine synthesized through HTGs is important for whitefly reproduction and fitness of both obligate and facultative symbionts, and it illustrates the mutual dependence between whitefly and its two symbionts. Collectively, this study reveals that acquisition of horizontally transferred lysine genes contributes to coadaptation and coevolution between *B*. *tabaci* and its symbionts.

## Author summary

Revealing which molecules are exchanged between host and symbionts will illuminate how host and bacterial symbiont establish beneficial relationships. Horizontally transferred genes (HTGs) presumably complement the missing genes involved in synthesis of essential amino acids (EAAs) in symbionts of sternorrhynchan insects. We demonstrate that whitefly HTGs can cooperate with *Portiera* and *Rickettsia* for lysine synthesis. The lysine synthesized through HTGs impacts both whitefly reproduction and symbiont fitness. Taken together, we reveal that horizontally acquired lysine genes underlie the basis for the mutual dependence between whitefly and two intracellular symbionts. Therefore, our study demonstrates the critical role of HTGs in the synthesis of EAAs and their function in insect-symbiont interactions and coevolution.

## Introduction

Microbial symbionts in insects can help them utilize food with unbalanced nutritional content by providing specific nutrients that hosts cannot synthesize [1–5]. These symbionts are considered to be obligate symbionts. The role of obligate symbionts in providing essential amino acids (EAAs) has been convincingly demonstrated in Hemiptera species such as aphids feeding on plant phloem deficient in essential nutrients [6]. Other symbionts associated with insects, which may affect insect fitness under certain conditions, are called facultative symbionts [6]. Many insect symbionts are specifically localized in the gut and hemocoel or within specialized host cells (bacteriocytes) [6]. Genome reduction is typical in intracellular symbionts, particularly of those required by hosts and that are restricted to bacteriocytes [7]. To maintain the benefits of symbiosis, host insects must adapt to support and control symbionts. For example, some host genes are enriched in bacteriocytes and they can complement the missing genes involved in synthesis of essential metabolites by the symbiont in the aphid, mealybug, psyllid and whitefly [1, 7–9]. The sophisticated metabolic integration between host and symbiont is a characteristic signature of host-symbiont coevolution [7, 10]. Additionally, the symbiont *Sodalis pierantonius* housed in bacteriocytes provides tyrosine and phenylalanine for the cereal weevil to build its exoskeleton. Once the cuticle is achieved, the symbiont is eliminated by host apoptosis and autophagy activation [11]. However, our understanding of insect-symbiont coadaptation is limited [7].

Horizontal gene transfer is the asexual transmission of genetic information between reproductively isolated species and has great impacts on genomic evolution [12]. Horizontally transferred genes (HTGs) that have originated from bacteria are known to be prevalent among prokaryotes. HTGs are also being increasingly reported in arthropod herbivores, and are widespread in insect symbiosis [1, 8–10]. In aphids, horizontally transferred *RlpA4* encodes a protein that is transported to the symbiont *Buchnera* [13]. Silencing horizontally transferred *amiD* and *ldcA1* decreases the abundance of *Buchnera* [14]. Some HTGs appear to be involved in synthesis of important metabolites in the insect-symbiosis system [7]. For example, HTGs in the mealybug genome cooperate with the symbiont *Moranella* for peptidoglycan synthesis [15]. Horizontally transferred whitefly biotin genes of bacterial origin can synthesize biotin [16]. Mealybug, psyllid and whitefly studies suggest that HTGs can complement the missing genes involved in synthesis of multiple EAAs in symbionts [1, 8, 9]. However, the role of HTGs in the synthesis of EAAs and their function in insect-symbiont interactions and coevolution remain largely unknown.

The whitefly *Bemisia tabaci* is a complex of more than 40 cryptic species as revealed by phylogenetic analyses and mating experiments [17, 18]. *B*. *tabaci* MEAM1 is one of the most important and invasive pests of agriculture [19, 20]. The whitefly *B*. *tabaci*-bacteria symbiosis is a valuable model system. All *B*. *tabaci* species harbor the obligate symbiont ‘*Candidatus* Portiera aleyrodidarum’ (hereafter *Portiera*) in bacteriocytes. *B*. *tabaci* also harbor up to four facultative symbiont lineages out of seven bacterial genera [21, 22]. The whitefly *B*. *tabaci* MEAM1 harbors *Portiera* and ‘*Candidatus* Hamiltonella defensa’ (hereafter *Hamiltonella*) in the same bacteriocyte and *Rickettsia* spp. (hereafter *Rickettsia*) in the whole body cavity [16]. These three symbionts are vertically transmitted via the egg [23, 24]. *Portiera* and *Hamiltonella* are fixed and *Rickettsia* has high infection frequencies (up to 100% depending on the geographical location) in the population of *B*. *tabaci* MEAM1 in China [23–26]. The genome of *Portiera* is highly reduced but it maintains genes involved in synthesis of ten EAAs [27, 28]. In contrast, the genomes of *Hamiltonella* and *Rickettsia* are moderately degenerated and only have some genes involved in synthesis of a few EAAs [29]. *Hamiltonella* can affect the *B*. *tabaci* sex ratio by facilitating fertilization and provisioning of five B vitamins [25]. In the USA, the increase of *Rickettsia* infection in *B*. *tabaci* MEAM1 populations from 2000 to 2011 conferred whitefly fitness benefits [30, 31] but the mechanism involved remains unknown. Horizontally transferred *dapB*, *dapF* and *lysA* with the phylogenetic origin of Rickettsiales, Enterobacteriales and Planctomycetes, respectively, are encoded in the genome of *B*. *tabaci* MEAM1 and highly expressed in bacteriocytes [9, 29]. It is likely these genes are involved in lysine synthesis. However, how these horizontally transferred lysine genes contribute to interactions and the coevolution of *B*. *tabaci* and symbionts is unclear. In this study, the function of horizontally transferred lysine genes in the *B*. *tabaci* MEAM1-tripartite symbiosis system was investigated. We reveal that lysine produced by *B*. *tabaci* HTGs affects the fecundity of whiteflies and the fitness of *Portiera* and *Rickettsia*.

## Results

### The lysine synthesis pathway in the tripartite symbiosis in *B*. *tabaci* MEAM1

**Fig 1:**
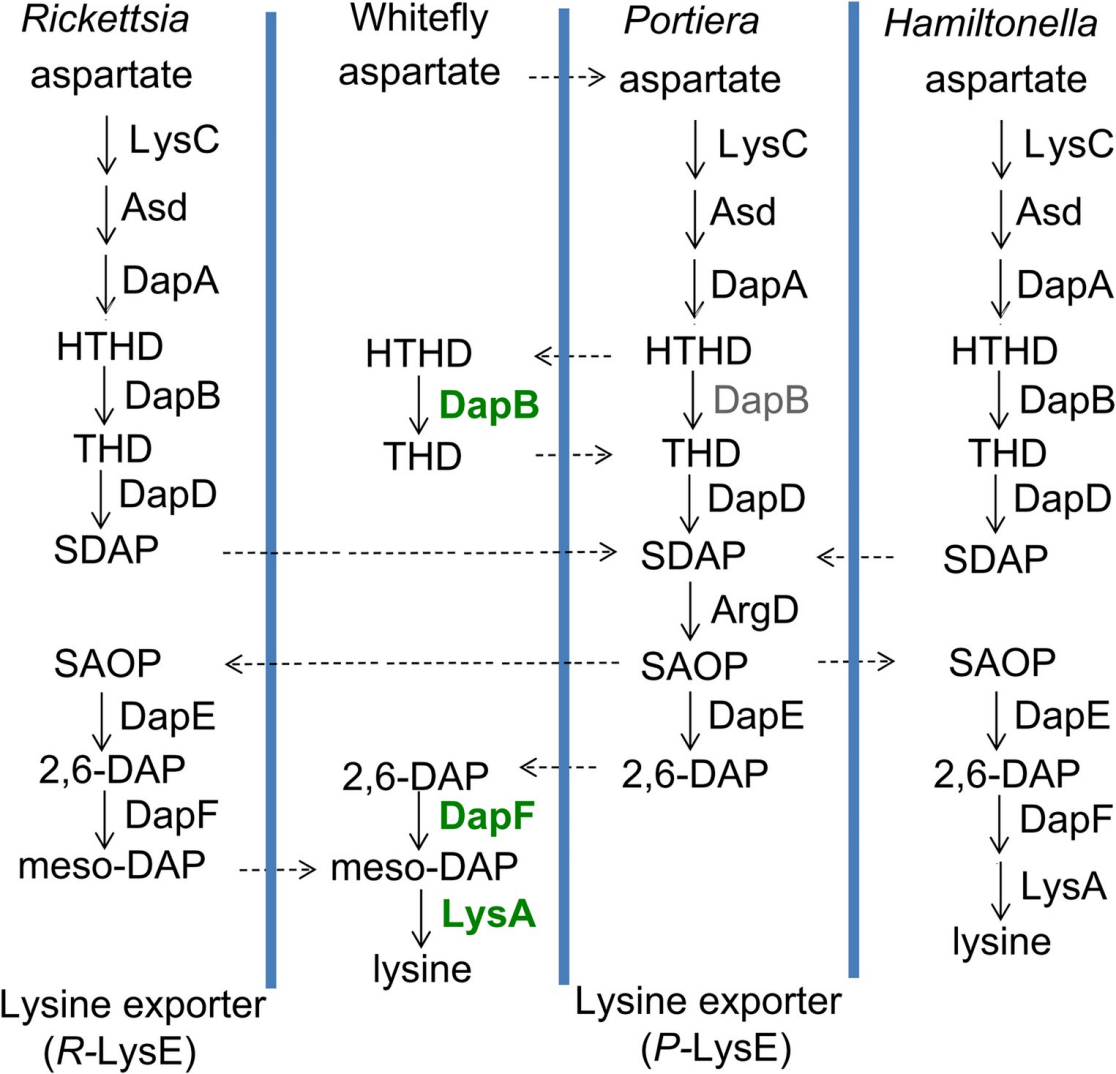
Lysine synthesis pathway in *B*. *tabaci* MEAM1. This figure is adapted from previous work (9, 29). HTHD, 4-hydroxy-2,3,4,5-tetrahydrodipicolinate; THD, 2,3,4,5-tetrahydrodipicolinate; SDAP, N-succinyl-L-2,6-diaminoheptanedioate; SAOP, N-succinyl-L-2-amino-6-oxoheptanedioate; 2,6-DAP, 2,6-diaminopimelate; meso-DAP, meso-2,6-diaminopimelate. Pseudogene is in grey and enriched host genes of bacterial origin are in green. The exchanges of intermediates among *Portiera*, *Hamiltonella* and *Rickettsia* are speculated.

Although the genomes of *Portiera*, *Hamiltonella*, and *Rickettsia* are reduced to varying degrees, these three symbionts possess most of the genes involved in lysine synthesis (S1 and S2 Data). In particular, *Rickettsia* only retains the synthesis pathway for one essential amino acid lysine (S1 Data). The *Rickettsia* genome lacks *lysA*, and the *Portiera* genome lacks *dapF* and *lysA* and has the pseudogene *dapB* for lysine synthesis (Fig 1 and S1 and S2 Data). In contrast, the *Hamiltonella* genome maintains an almost intact lysine synthesis pathway except that it lacks *argD*, which may need complementation by *Portiera* (Fig 1 and S2 Data). The horizontally transferred *dapB*, *dapF*, and *lysA* in *B*. *tabaci* MEAM1 seem to be able to compensate for the missing genes in both *Portiera* and *Rickettsia* (Fig 1). The lysine exporter family protein LysE is present in both *Portiera* (Por0095) and *Rickettsia* (Ric0176) but absent from *Hamiltonella* (Fig 1) [29], indicating that both *Portiera* and *Rickettsia* are able to transport lysine for whiteflies.

### Evolutionary origin of horizontally transferred lysine genes in whiteflies

Our previous work has shown that horizontally transferred *dapB*, *dapF* and *lysA* were detected in the transcriptomes of *B*. *tabaci* MEAM1, MED and Asia II 3, and that they could be assigned to Rickettsiales, Enterobacteriales and Planctomycetes, respectively [9, 29]. To determine if horizontally transferred lysine genes are ubiquitous in whitefly populations, the presence of *dapB*, *dapF* and *lysA* was checked in multiple whitefly species and cultures. We found that *dapB* and *dapF* have a single copy in the genome of *B*. *tabaci* MEAM1 that lacks an intron [29]. By contrast, *lysA* was duplicated in the genome of *B*. *tabaci* MEAM1 [29]. These two *lysA* genes (Bta03593 and Bta03589) were located in the positive strand and negative strand of the whitefly genome, respectively [29]. Bta03593 has acquired introns while Bta03589 was largely truncated and lacked the essential pyridoxal 5’-phosphate binding site, catalytic residues, and substrate binding site (S1A–S1C Fig). Thus, the gene sequence of *lysA* Bta03593 was used in the following analysis and experiments, if not otherwise specified. The horizontally transferred lysine genes were present in 13 whitefly cultures of ten *B*. *tabaci* cryptic species, originating from Asia, America, and Africa, but absent from a phylogenetically-distant whitefly species, *Trialeurodes vaporariorum* (S1 Table). The data suggest that acquisition of horizontally transferred lysine genes may have occurred after whiteflies diverged into *Bemisia* and *Trialeurodes*. In *B*. *tabaci* MEAM1, *Hamiltonella* has *dapB*, *dapF*, and *lysA* and *Rickettsia* has *dapB* and *dapF* (S1 and S2 Data). To examine the divergence of protein sequences, amino acid sequences were aligned among ten whitefly cryptic species, as well as *Hamiltonella* and *Rickettsia* of *B*. *tabaci* MEAM1 for DapB, DapF and LysA. As only transcriptome data for *B*. *tabaci* Asia II-3 is available, and the sequencing depth and coverage for Asia II-3 is not high enough, the obtained amino acid sequences for DapB, DapF and LysA in this species are shorter compared to the other nine *B*. *tabaci* species. Whereas, the amino acid sequence identity was high among all ten whitefly *B*. *tabaci* cryptic species (91.9% for DapB, 82.1% for DapF and 97.1% for LysA), and it was low between *B*. *tabaci* MEAM1 and *Hamiltonella* (31.5% for DapB, 45.4% for DapF and 22.07% for LysA) and *B*. *tabaci* MEAM1 and *Rickettsia* (46.1% for DapB and 26.8% for DapF) (S2–S4 Figs). These data suggest that these whitefly lysine genes are not likely to have been horizontally transferred from *Hamiltonella* and *Rickettsia*. Moreover, catalytic sites are conserved in all ten *B*. *tabaci* cryptic species. To gain insight into the evolution of these HTGs, a phylogenetic tree was constructed. Interestingly, the DapB, DapF and LysA of all whitefly *B*. *tabaci* cryptic species clustered within the same clade (S5–S7 Figs), suggesting that horizontally transferred lysine genes share a common evolutionary origin in all whitefly *B*. *tabaci* cryptic species. Whitefly DapB fell within the clade of *Rickettsia* and clustered with the *Rickettsia* symbiont of *Culicoides newsteadi*, DapF clustered with *Pantoea* and bacterial symbiont of *Plautia stali*, and LysA clustered with Planctomycetes (S5–S7 Figs).

### Effect of *Hamiltonella* deficiency on lysine gene expression, lysine level and protein localization in bacteriocytes and ovaries of whiteflies

Whitefly lifestages consist of egg, nymphs and adult. Adult females were used for all trials in this study as this was not only more convenient experimentally but also made sense from a biological perspective. Female adult whiteflies can lay hundreds of eggs in their lifespan [32] and the level of hemolymph vitellogenin (vg) accumulates in the developing oocytes providing nutrition for ovary development during oogenesis [33–35]. High levels of EAAs including lysine and others are required for vg synthesis in female adult whiteflies.

**Fig 2:**
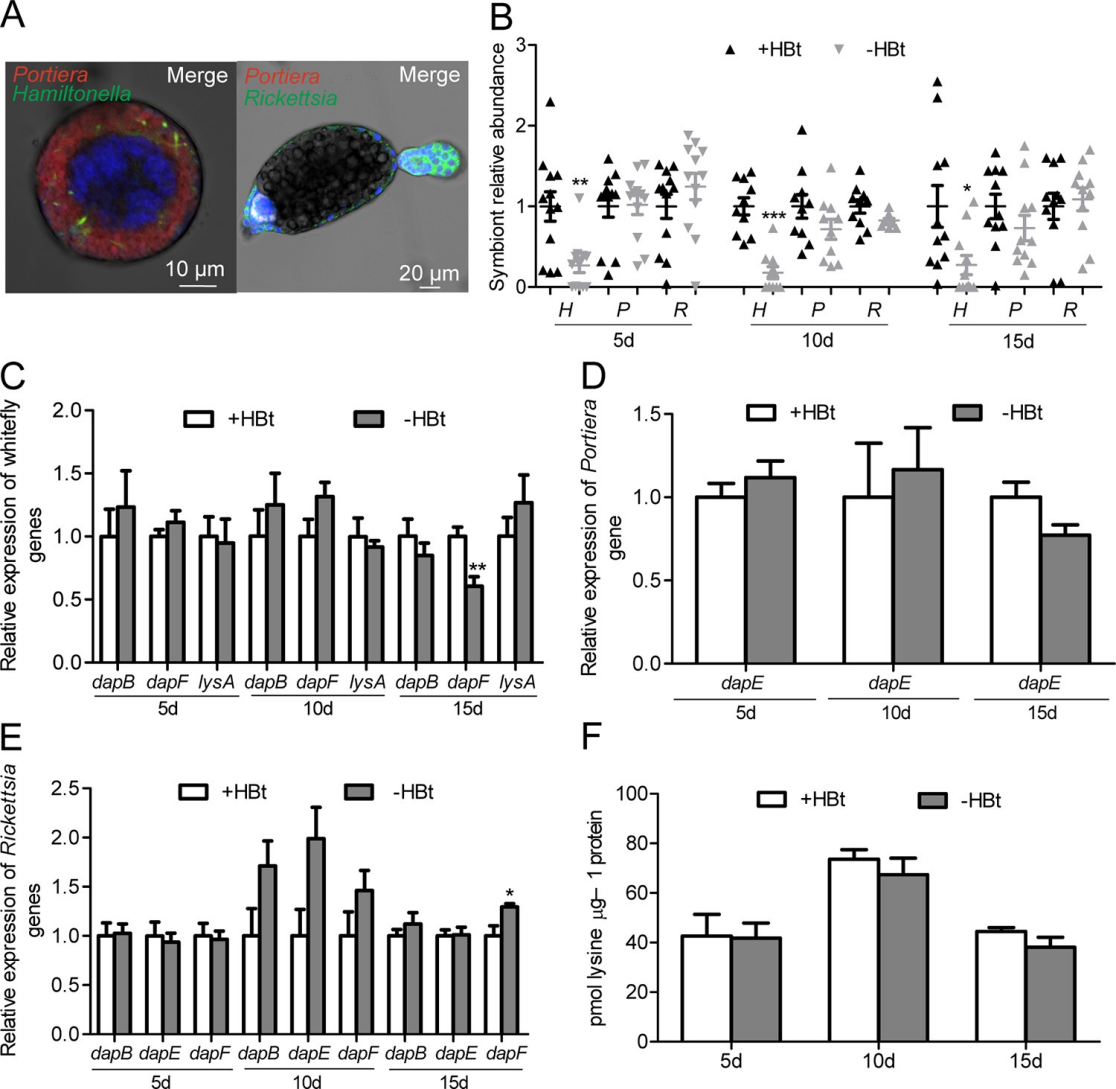
Effects of *Hamiltonella* deficiency on lysine gene expression and lysine levels in *B*. *tabaci*. (A) Localization of symbiotic bacteria *Portiera* (red) and *Hamiltonella* (green) in the whitefly bacteriocyte, as well as *Portiera* (red) and *Rickettsia* (green) in the whitefly ovary. n = 3. (B) Effects of antibiotic treatments on the abundance of symbionts in *B*. *tabaci* at 5 d, 10 d, and 15 d after emergence. *H*, *P* and *R* represent *Hamiltonella*, *Portiera* and *Rickettsia*, respectively. n = 12, 10 and 11 for 5 d, 10 d, and 15 d, respectively. (C) Effects of *Hamiltonella* deficiency on expression of whitefly lysine genes in *B*. *tabaci* at 5 d, 10 d, and 15 d after emergence. n = 4. (D) Effects of *Hamiltonella* deficiency on expression of *Portiera* lysine gene in *B*. *tabaci* at 5 d, 10 d, and 15 d after emergence. n = 4. (E) Effects of *Hamiltonella* deficiency on expression of *Rickettsia* lysine genes in *B*. *tabaci* at 5 d, 10 d, and 15 d after emergence. n = 4. (F) Effects of *Hamiltonella* deficiency on lysine levels in *B*. *tabaci* at 5 d, 10 d, and 15 d after emergence. n = 6. +HBt and -HBt represent *Hamiltonella*-infected and *Hamiltonella*-cured whiteflies, respectively. Data are means ± SEM. The significant differences between treatments are indicated by asterisks (*P < 0.05; **P < 0.01; ***P < 0.001).

*Portiera* and *Hamiltonella* are housed in bacteriocytes and ovaries and *Rickettsia* in ovaries, guts and other body tissues except for bacteriocytes of *B*. *tabaci* MEAM1 (Fig 2A) [24]. After antibiotic cocktail treatment, *Hamiltonella* was reduced by 72.7–82.2% and the abundance of *Portiera* and *Rickettsia* remained unchanged in whiteflies at 5, 10 and 15 d after emergence at the F1 adult stage (Fig 2B; *P* < 0.05 for *Hamiltonella*; *P* > 0.05 for *Portiera* and *Rickettsia*). After the titer of *Hamiltonella* was reduced, expression of whitefly *dapB*, *dapF* and *lysA* remained unchanged at 5, 10 and 15 d after emergence at the F1 adult stage after antibiotic treatment except for *dapF* at 15 d after emergence (Fig 2C; *P* > 0.05 for *dapB* and *lysA* at 5 d, 10 d, and 15 d and *dapF* at 5 d and 10 d; *P* = 0.0098 for *dapF* at 15 d). After *Hamiltonella* titer was reduced, expression of *dapE* of *Portiera*, as well as *dapB*, *dapE* and *dapF* of *Rickettsia* remained unchanged at 5, 10 and 15 d after emergence at the F1 adult stage after antibiotic treatment except for *dapF* at 15 d after emergence (Fig 2D and 2E; *P* > 0.05 for *dapE* of *Portiera*, *dapB* and *dapE* of *Rickettsia* at 5, 10 and 15 d and *dapF* of *Rickettsia* at 5 and 10 d; *P* < 0.05 for *Rickettsia dapF* at 15 d).

Our previous UPLC analyses showed that elimination of *Hamiltonella* did not influence the lysine level in the whole body of adult *B*. *tabaci* [25]. To determine if *Hamiltonella* is involved in lysine provisioning, *Hamiltonella* were specifically cured by antibiotic treatments and lysine levels were measured over time. *Hamiltonella* deficiency did not significantly change the lysine level in the whole body of adult *B*. *tabaci* feeding on cotton plants at 5 d, 10 d, and 15 d after emergence at the F1 adult stage after antibiotic treatment (Fig 2F; *P* = 0.17–0.94).

**Fig 3:**
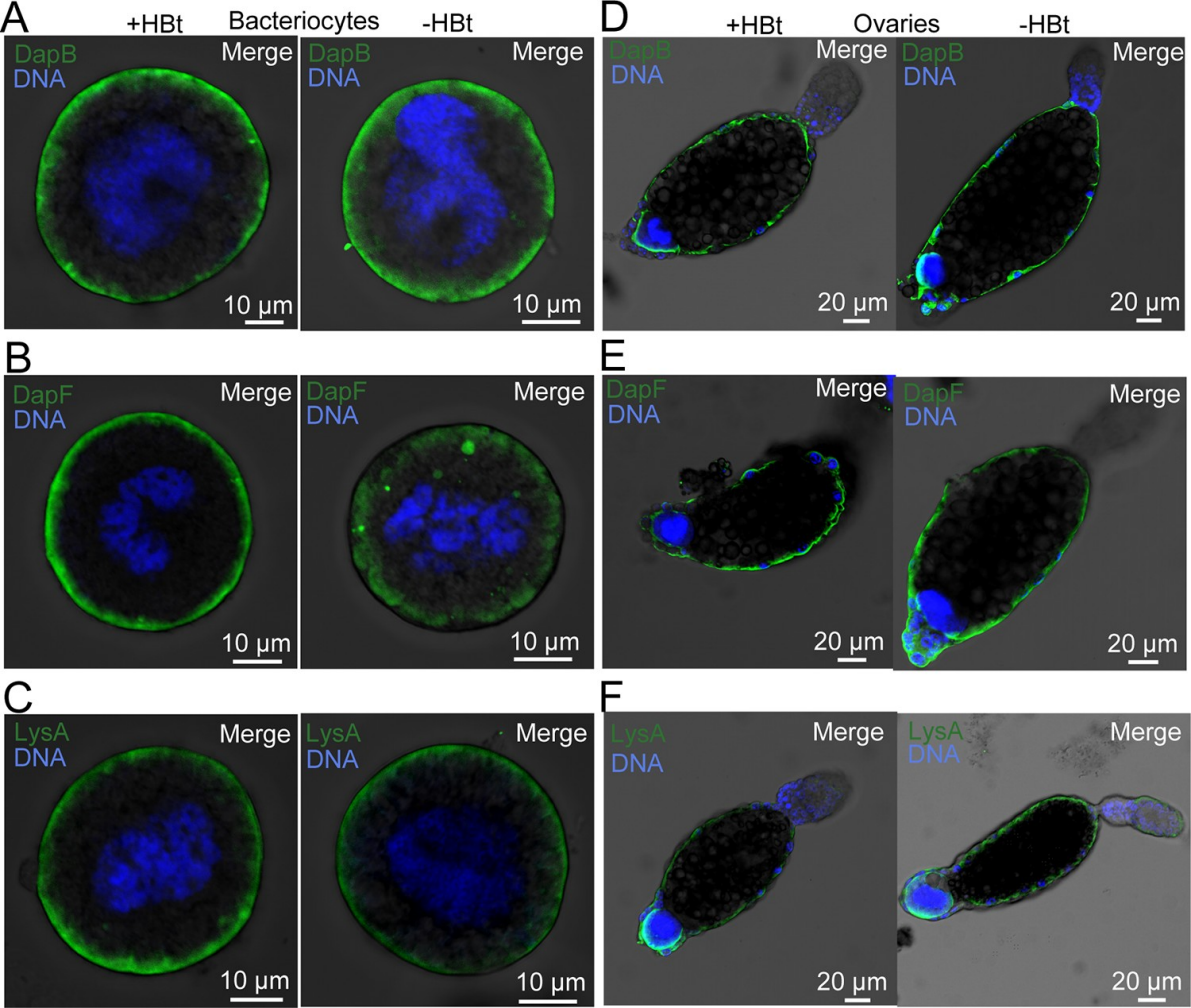
Effects of *Hamiltonella* deficiency on protein localization in *B*. *tabaci*. Localization of DapB, DapF and LysA proteins (green) in bacteriocytes (A-C) and ovaries (D-F) of female adult whiteflies. DNA was stained with DAPI. +HBt and -HBt represent *Hamiltonella*-infected and *Hamiltonella*-cured whiteflies, respectively. n = 3.

To examine the subcellular location of the proteins encoded by whitefly *dapB*, *dapF* and *lysA*, recombinant proteins were successfully generated (S8A–S8C Fig). Then, polyclonal antibodies against DapB, DapF and LysA proteins were produced using the purified recombinant protein. The polyclonal antibodies had good specificity, which was verified by western blot (S8D–S8F Fig). Immunofluorescence microscopy showed that DapB, DapF, and LysA were mainly located in the peripheral regions of bacteriocytes (Fig 3A–3C) as well as in the follicle cells and bacteriocytes of ovaries (Fig 3D–3F). After *Hamiltonella* was cured, the protein expression levels and patterns were maintained in whitefly bacteriocytes as well as ovaries (Fig 3A–3F), confirming that DapB, DapF, and LysA were not encoded by *Hamiltonella*. There was no signal of DapB, DapF, and LysA in whitefly bacteriocytes and ovaries of negative controls (S9 Fig). Because the *Rickettsia* genome lacks *lysA*, the presence of LysA in the follicle cells of ovaries is not due to *Rickettsia*, which infects whitefly ovaries (Fig 2A). Likewise, after *Portiera*, *Rickettsia* and *Hamiltonella* were reduced by 98%, 88%, and 98%, respectively (S10A Fig; *P* < 0.01), the LysA protein expression levels and patterns were maintained in whitefly guts (S10B and S10C Fig), further confirming that LysA was not encoded by *Rickettsia*, which infects whitefly guts [24].

### Functional complementation of *E*. *coli* lysine auxotrophs with whitefly lysine genes

**Fig 4:**
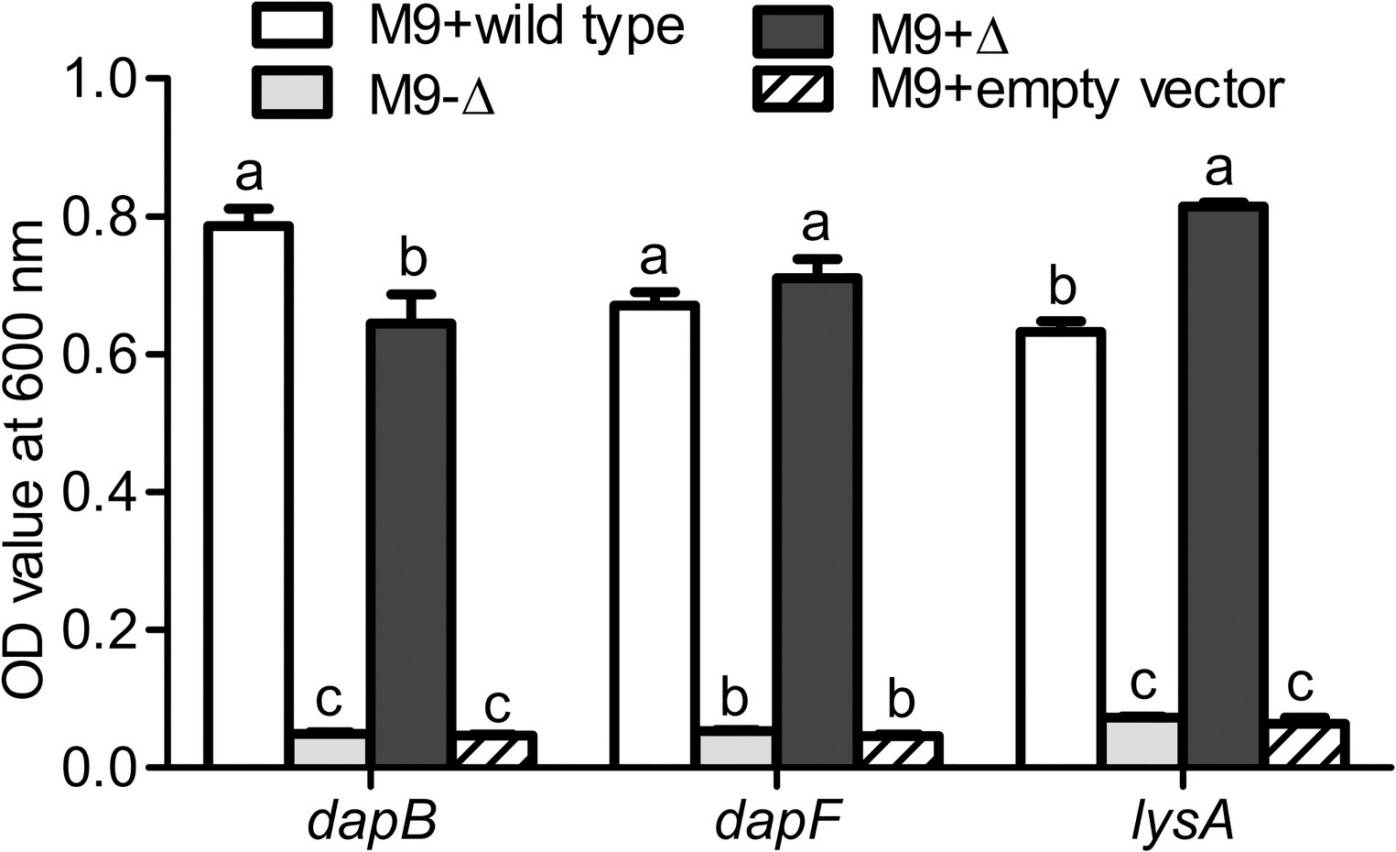
Functional complementation of *E*. *coli* lysine auxotrophs. *E*. *coli* K-12 knockout mutant cells were transformed with expression plasmids containing whitefly *dapB*, *dapF*, and *lysA* or the negative control pMD19-T empty vector. The *E*. *coli* wild-type K-12, mutant K-12 (-Δ) and mutant K-12 transformants (+Δ) were grown overnight in amino acid-deficient M9 liquid medium at 37°C. All *E*. *coli* cells were washed and re-suspended to measure cell density at OD_600_. Data are means ± SEM. n = 3. Different letters above the bars indicate significant differences between treatments at *P* < 0.05.

To test the hypothesis that whitefly *dapB*, *dapF*, and *lysA* function in lysine synthesis, the *E*. *coli* K-12 *dapB*, *dapF*, and *lysA* knockout mutant (-Δ*dapB*, -Δ*dapF*, or -Δ*lysA*) were generated using the Lambda Red protocol and functionally complemented *E*. *coli* K-12 mutant with whitefly *dapB*, *dapF*, and *lysA*, respectively. Compared to wild type *E*. *coli*, *E*. *coli* K-12 knockout mutants (-Δ*dapB*, -Δ*dapF*, and -Δ*lysA*) did not grow on M9 minimal medium lacking lysine (Fig 4). Although whitefly *dapB*, *dapF*, and *lysA* shared low amino acid sequence identities with *E*. *coli* homolog genes (33.33%, 59.55%, and 27.48%, respectively), complementation with whitefly *dapB*, *dapF*, and *lysA* rescued *E*. *coli* K-12 knockout mutants on M9 minimal medium (Fig 4). In contrast, cells transformed with the empty vector of pMD19-T did not grow on M9 minimal medium without lysine supplementation (Fig 4). Significant differences in OD values among treatments were detected (Fig 4; *P*< 0.001 for *dapB*, *dapF*, and *lysA*).

### Silencing horizontally transferred *lysA* in whiteflies infected with *Hamiltonella* reduces lysine level, whitefly fecundity and the titers of *Portiera* and *Rickettsia*

**Fig 5:**
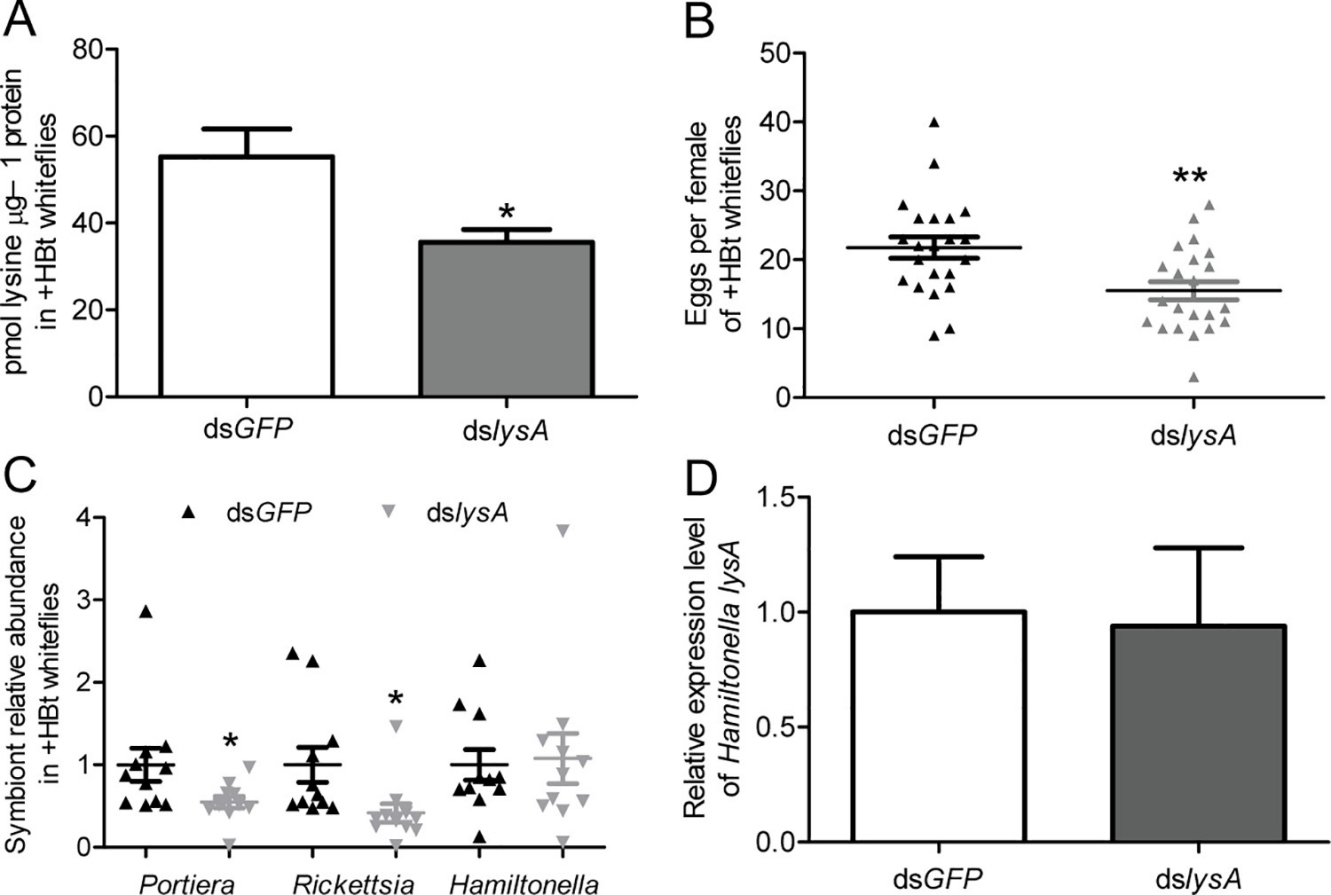
Effects of silencing horizontally transferred *lysA* on lysine levels, whitefly fecundity, symbiont titer and *Hamiltonella lysA* expression in whiteflies infected with *Hamiltonella*. (A) Lysine levels in whiteflies at day 3 after whiteflies were microinjected with ds*lysA*. n = 3. (B) Fecundity of female adult whiteflies at day 3 after microinjection with ds*lysA*. n = 22. (C) Effects of silencing horizontally transferred *lysA* on symbiont titer. n = 11. (D) Expression of *Hamiltonella lysA* at day 3 after whiteflies were microinjected with ds*lysA*. n = 4. ds*GFP* and ds*lysA* represent ds*GFP*-injected and ds*lysA*-injected female adult whiteflies, respectively. Data are means ± SEM. The significant differences between treatments are indicated by asterisks (*P < 0.05; **P < 0.01).

To confirm the metabolic function of horizontally transferred lysine genes, a gene silencing approach was applied in whiteflies infected with *Hamiltonella*. As the horizontally transferred *lysA* gene complements for the missing gene in both *Portiera* and *Rickettsia*, whitefly *lysA* was selected for silencing. The *lysA* nucleotide sequence identity was very low between *B*. *tabaci* MEAM1 and *Hamiltonella* (35.27%). A pair of primers was designed to specifically target *lysA* of the whitefly rather than *Hamiltonella* (S2 Table). Expression of whitefly *lysA* was reduced by 71% at day 3 after microinjection with dsRNAs (S11A Fig; *P* = 0.0089). After *lysA* gene silencing, the lysine level was significantly reduced by 35.6% in whiteflies (Fig 5A; *P* = 0.049). The fecundity of female adult whiteflies was significantly reduced at day 3 after microinjection with ds*lysA*, compared to that of whiteflies microinjected with ds*GFP* (Fig 5B; *P* = 0.0035). To evaluate the effect of *lysA* silencing on symbiont titer, the abundance of symbionts was quantified in *lysA* RNAi whiteflies. The abundance of *Portiera* and *Rickettsia* was reduced significantly at day 3 after whiteflies were microinjected with dsRNAs while the abundance of *Hamiltonella* did not change significantly (Fig 5C; *P* = 0.047 for *Portiera* and *P* = 0.024 for *Rickettsia*; *P* = 0.83 for *Hamiltonella*). Additionally, expression of *Hamiltonella lysA* remained unchanged at day 3 after microinjection with dsRNAs (Fig 5D; *P* = 0.9).

### Silencing horizontally transferred *lysA* in whiteflies lacking *Hamiltonella* reduces lysine level, whitefly fecundity and the titer of *Portiera* and *Rickettsia*

**Fig 6:**
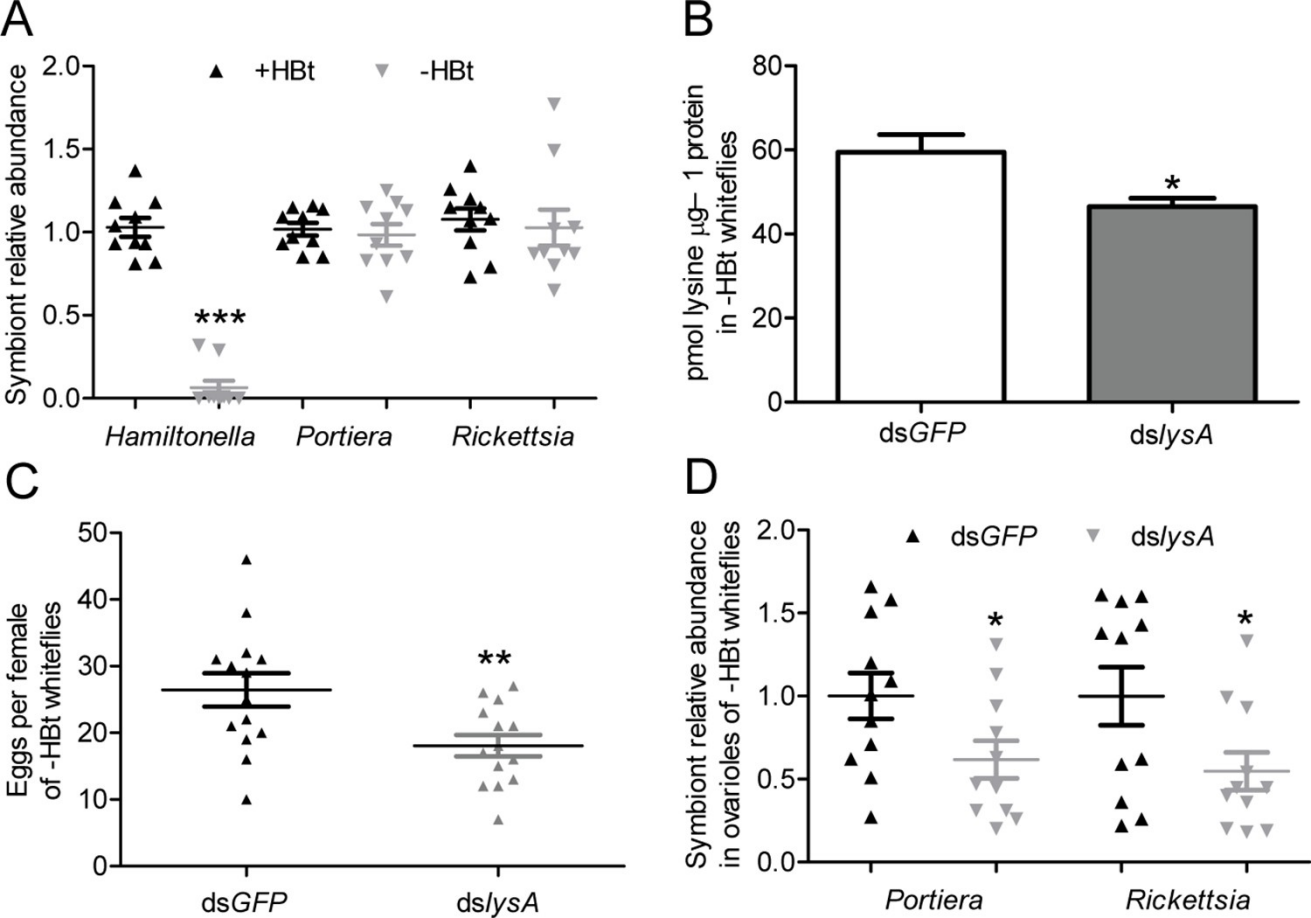
Effects of silencing horizontally transferred *lysA* on lysine levels, whitefly fecundity, and symbiont titer in whiteflies lacking *Hamiltonella*. (A) Effects of antibiotic treatments on the abundance of symbionts in *B*. *tabaci*. n = 10. (B) Lysine levels in *Hamiltonella*-cured whiteflies at day 3 after whiteflies were microinjected with ds*lysA*. n = 4. (C) Fecundity of *Hamiltonella*-cured female adult whiteflies at day 3 after microinjection with ds*lysA*. n = 14. (D) Effects of silencing horizontally transferred *lysA* on symbiont titer in ovarioles of *Hamiltonella*-cured whiteflies at day 3 after microinjection with ds*lysA*. n = 11. +HBt and -HBt represent *Hamiltonella*-infected and *Hamiltonella*-cured whiteflies, respectively. ds*GFP* and ds*lysA* represent ds*GFP*-injected and ds*lysA*-injected female adult whiteflies, respectively. Data are means ± SEM. The significant differences between treatments are indicated by asterisks (*P < 0.05; **P < 0.01; ***P < 0.001).

To further determine the role that *Hamiltonella* played in the reduced whitefly fecundity and symbiont titer after whitefly *lysA* silencing, the gene silencing was conducted in whiteflies lacking *Hamiltonella*. *Hamiltonella* was eliminated by 94% without influencing the abundance of *Portiera* and *Rickettsia* (Fig 6A; *P* < 0.0001 for *Hamiltonella*; *P* = 0.8 for *Portiera* and *P* = 0.97 for *Rickettsia*). Expression of whitefly *lysA* was significantly decreased by 77% at day 3 after microinjection with dsRNAs (S11B Fig; *P* = 0.00076). The lysine level was significantly reduced by 21.7% in *Hamiltonella*-cured whiteflies at day 3 after RNAi treatment (Fig 6B; *P* = 0.032). The fecundity of female adult whiteflies was significantly reduced at day 3 after microinjection with dsRNAs (Fig 6C; *P* = 0.0092). *Portiera* and *Rickettsia* are vertically transmitted in whiteflies via ovarioles. The changes of abundance of *Portiera* and *Rickettsia* in the whole body will influence the titer of *Portiera* and *Rickettsia* in ovarioles, thereby impacting the transmission of symbiont. Thus, whitefly ovarioles were collected for symbiont quantification after gene silencing. The abundance of *Portiera* and *Rickettsia* in ovarioles was reduced significantly at day 3 after whiteflies were microinjected with dsRNAs (Fig 6D; *P* = 0.044 for *Portiera* and *P* = 0.043 for *Rickettsia*). These data suggest that *Hamiltonella* did not contribute to the reduced lysine level, whitefly fecundity and symbiont titer in whiteflies after RNAi treatment.

## Discussion

**Fig 7:**
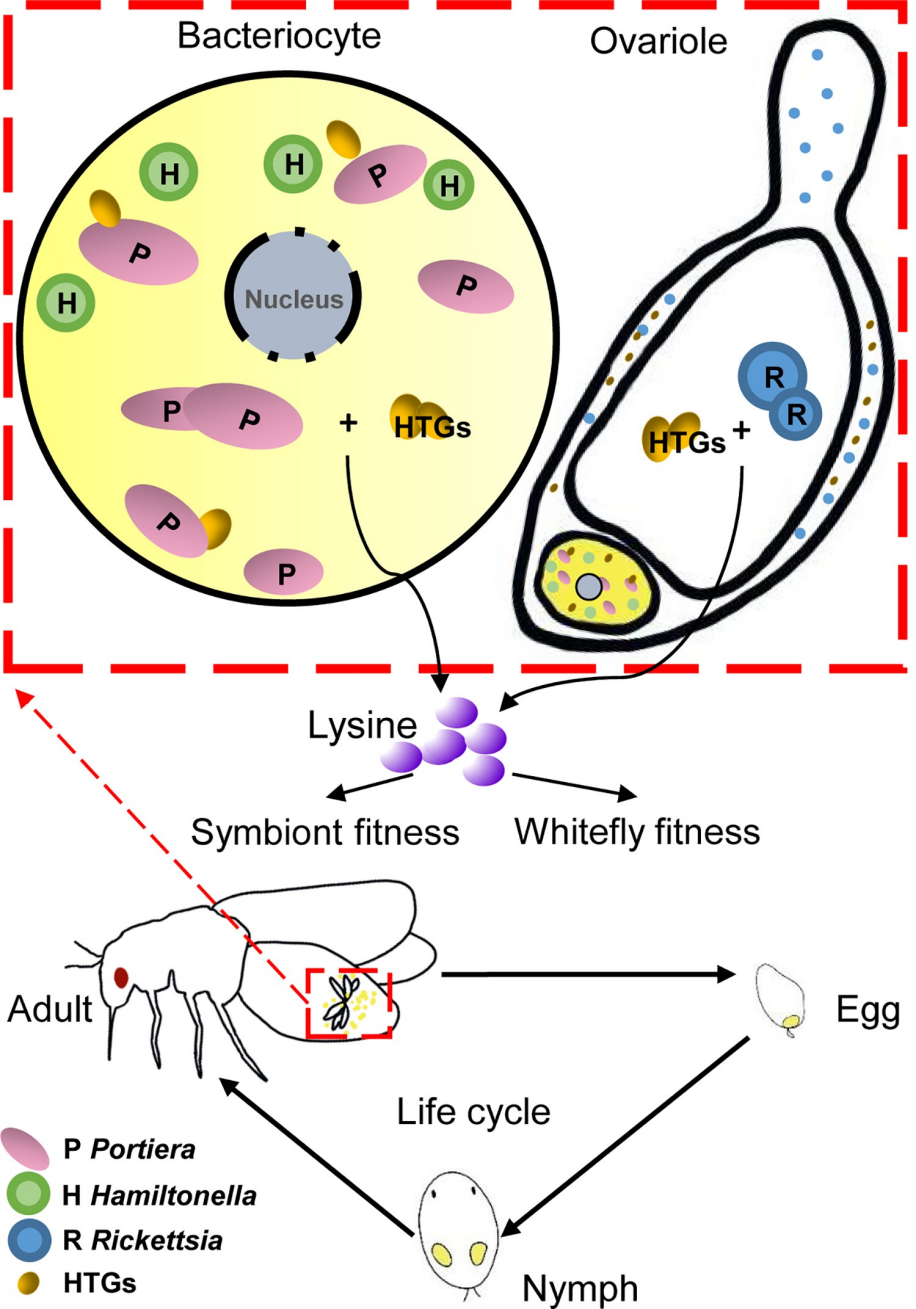
Schematic overview of how lysine provisioning by horizontally acquired bacteria genes promotes mutual dependence between whitefly and two intracellular symbionts. The whitefly lifestages consist of egg, nymphs and adult. Whiteflies can synthesize lysine through the cooperation of horizontally transferred genes (HTGs) and two symbionts (one obligate symbiont *Portiera* and one facultative symbiont *Rickettsia*). The lysine synthesized through HTGs impacts the fecundity of adult whiteflies and titers of two symbionts.

This study reveals that lysine HTGs underlie the mutual dependence between whitefly and two of its symbionts in a tripartite symbiosis (Fig 7). *Portiera* as an obligate symbiont is known to be required for survival of *B*. *tabaci* [36, 37]. Similarly increases in infection frequencies with the facultative symbiont *Rickettsia* have for MEAM1 *B*. *tabaci* populations in USA from 2000–2011 been linked to improved fitness [30, 31]. The high *Rickettsia* infection frequencies in the populations of *B*. *tabaci* MEAM1 in China from 2011–2014 [24], may be offering similar benefits. It is the first to demonstrate the key role of these lysine HTGs in *B*. *tabaci* reproduction as well as fitness of both its obligate symbiont *Portiera* and facultative symbiont *Rickettsia*. As an EAA, lysine plays critical roles in protein synthesis in all living organisms [6, 38]. There are, however, generally only low levels of EAAs including lysine in the phloem of various plant species [6] posing a nutritional challenge for phloem-feeding insects. The localization of whitefly DapB, DapF and LysA in bacteriocytes/ovaries facilitates the cooperation of the whitefly and *Portiera*/*Rickettsia* (respectively) for lysine synthesis. This study shows that whiteflies have acquired lysine HTGs of bacterial origin which synthesize lysine through the cooperation with two symbionts, which thus benefits whitefly fitness.

Almost complete elimination of the facultative symbiont *Hamiltonella* was found in this study to not influence lysine levels in whiteflies over time. This indicates that *Hamiltonella* may not synthesize lysine or may synthesize only a low amount of lysine for its own benefits. If *Hamiltonella* can synthesize lysine, reduced lysine in *Hamiltonella-*cured whiteflies might not have had an effect due to being complemented for by lysine synthesized by the cooperation between whitefly and *Portiera*/*Rickettsia*. As such, lysine levels could have appeared unchanged in *Hamiltonella-*eliminated whiteflies over time. There was however no influence of *Hamiltonella* deficiency on the abundance of *Portiera* and *Rickettsia*, and no associated effect on the expression of lysine genes of whiteflies, *Portiera* and *Rickettsia*, nor localization of horizontally transferred lysine protein. *Hamiltonella* deficiency also did not impact whitefly *lysA* silencing on lysine level, whitefly performance and symbiont abundance. Moreover, expression of *Hamiltonella lysA* remained unchanged after whitefly *lysA* RNAi, which excludes the potential dsRNA non-target effects for *Hamiltonella lysA*. Thus, the reduced lysine levels, whitefly fecundity and titers of *Portiera* and *Rickettsia* by silencing *lysA* cannot be attributed to any role of *Hamiltonella* in lysine production for whiteflies.

All 13 cultures or colonies from ten *B*. *tabaci* cryptic species were shown to possess lysine HTGs. Phylogenetic analyses revealed that whitefly DapB clusters with *Rickettsia*, DapF clusters with *Pantoea* and bacterial symbionts of *P*. *stali*, and LysA clusters with Planctomycetes. The lysine HTGs in different *B*. *tabaci* species from diverse geographical regions thus appear to share a common evolutionary origin. *Rickettsia* is present in *B*. *tabaci* MEAM1, Asia II 3, and MED [16, 22, 39, 40]. So, *dapB* is likely to have been transferred to a common ancestor of *B*. *tabaci* from *Rickettsia*. It may be that *Pantoea* and bacterial symbionts of *P*. *stali* as well as Planctomycetes were historical symbionts or pathogens of *B*. *tabaci*, providing the sources of horizontally transferred DapF and LysA, respectively, for whiteflies. In contrast with *B*. *tabaci*, lysine HTGs are not found in *T*. *vaporariorum*. The retention of an intact lysine synthesis pathway for *Portiera* in *T*. *vaporariorum* [28] supports the absence of lysine HTGs. It appears probable therefore that bacterial genes were transferred to the common ancestor of the *B*. *tabaci* species studied, which in turn facilitated the loss of genes in *Portiera* [27, 28].

Previously, we have identified redundancy in the arginine synthetic pathway by HTGs that was not needed by their symbionts, possibly “promoting” dependence on the host in *B*. *tabaci* MEAM1 and MED. It is the horizontally transferred *argH* involved in arginine synthesis in whiteflies, while *argH* is a pseudogene in *Portiera* [9]. Both *dapF* and *argH* clustered with *Pantoea* and bacterial symbionts of *P*. *stali* [9]. Thus, *dapF* and *argH* could have been acquired from *Pantoea* or close relatives of gut symbionts of the stinkbug *P*. *stali*. In contrast, *dapB* and *lysA* from different bacteria may have been independently acquired in parallel. After being horizontally transferred to the whitefly genome, these lysine genes evolved to become functional. The best example is *lysA* which has duplicated genes in the genome of *B*. *tabaci* MEAM1 [29]. One *lysA* has acquired an intron whereas the other is largely truncated. This *lysA* case provides evidence that intron gain and duplication of HTGs are critical steps for attaining functionality in a eukaryotic genome [41]. By contrast, *dapB* and *dapF* have no introns suggesting they may have been horizontally acquired relatively recently compared to *lysA* in whiteflies.

In newly emerged young adult whiteflies, oogenesis happens very frequently [33, 35], and a high level of lysine is required for oogenesis, as in other animals [42–44]. As lysine is one of the top limiting EAAs [45], reduction of even small levels of lysine impacts animal phenotype, particularly during oogenesis that requires lots of nutrients [42–44]; *lysA* is the terminal gene in the lysine synthesis pathway. Thus, it is reasonable that silencing *lysA* in female adult whiteflies inhibits lysine production, whitefly fecundity and symbiont fitness. Further investigation on the kinetics of lysine catabolism and anabolism in whiteflies will facilitate the study on insect nutritional physiology. It will also help us to understand better the role of lysine in insect symbiosis.

There is redundancy in lysine synthesis in *B*. *tabaci*, *Portiera*, *Hamiltonella* and *Rickettsia*. However, the lysine synthesis pathway in the three symbionts has degenerated at different levels. It seems likely that the main function of *Portiera* in lysine synthesis can be streamlined into a few genes among *lysC*-*dapA* and *argD*-*dapE*. In the lysine synthesis pathway of *Portiera*, *dapB* and *dapD* have significantly lower expression levels compared to *dapA* and *dapE* [9]. The differentiated abundance of transcripts can lead to the further loss of genes such as *dapD* in *Portiera*, which may depend on facultative symbionts, or promote novel functionalization of HTGs that could be more beneficial than harboring a whole *Rickettsia* symbiont.

Lysine HTGs in *B*. *tabaci* species may facilitate the loss of lysine synthesis capability of its symbionts *Hamiltonella* and *Rickettsia*. Likewise, the lysine synthesis pathways of *Moranella* and *Tremblaya* are degenerated in the mealybug *Planococcus citri*, which possesses lysine HTGs (*dapF* and *lysA*) with *Rickettsiales* origin [1]. These findings suggest the parallel evolution of horizontal gene transfer has occurred facilitating reduction of lysine synthesis capability of symbionts in phloem-feeding insects.

The titer of *Portiera* and *Rickettsia* but not *Hamiltonella* was reduced in whiteflies over a 3 day period after silencing *lysA*. There are two reasons. First, a lower titer of *Hamiltonella* in whiteflies as compared to *Portiera* and *Rickettsia* has been reported [16, 24, 25]. After silencing *lysA*, sufficient lysine levels may be still present in the whitefly to not have a large effect on the titer of *Hamiltonella* over short time periods. Second, although it is speculated that *Hamiltonella* lacks ArgD for lysine synthesis and may require an intermediate from *Portiera*, some proteins may be promiscuous in the reduced *Hamiltonella* genome as reported earlier for *Buchnera* [46] and functionally replace ArgD. As such, *Hamiltonella* may encode the full lysine synthesis pathway. The loss of ArgD in the symbionts of both mealybug and psyllid also suggests such a possibility [1, 8].

Determining what molecules are exchanged between host and symbionts increases our understanding of how hosts support symbionts [7]. The genomes of *Portiera*, *Hamiltonella* and *Rickettsia* contain the lysine synthesis pathway [9, 27, 29, 47], suggesting the critical role of lysine in the biology of three symbionts and their interactions with whiteflies. *Portiera*, *Hamiltonella* and *Rickettsia* are vertically transmitted in whiteflies [23, 24, 48, 49]. Silencing horizontally transferred *lysA* decreased whitefly fecundity, which would reduce the transmission of the three symbionts. Additionally, silencing horizontally transferred *lysA* decreased the titer of *Portiera* and *Rickettsia* in either whole body or ovarioles. Thus, regulation on lysine HTGs in whiteflies controls the fitness and transmission of its intracellular symbionts. Further study on (i) the flux of lysine via labeled amino acid experiments, (ii) lysine stability and kinetics in the whitefly body and tissues, (iii) the transport of lysine between whitefly cells and symbiont cells and (iv) lysine regulation of symbiont proliferation should provide interesting insights of how whiteflies ensure symbionts remain at beneficial levels. Improved knowledge on these pathways will also assist selection of which genes would make the best targets to silence for the control of *B*. *tabaci* species, many of which are important and invasive pests affecting food security.

Previously, we revealed the function of HTGs in the synthesis of B vitamins in whiteflies [16, 50]. Here, we, for the first time, demonstrated that an EAA lysine synthesis, by the cooperation of lysine HTGs and *Portiera*/*Rickettsia*, promotes mutual dependence between whitefly and two intracellular symbionts (both obligate and facultative symbionts) (Fig 7). The function of each horizontally acquired gene differs, depending on its encoded enzyme type, metabolite function, metabolite synthesis pathway, and the species involved in the actual insect-symbiont system [7, 13–16]. Moreover, the functional significance of HTGs in the field is still quite scarce and developing. Therefore, it will be valuable to investigate the function of every HTG in diverse insect-symbiont systems to gain more insight into the evolutionary and functional significance of HTGs in insect-symbiont interactions in general.

## Materials and methods

### Insect rearing and plants

The *B*. *tabaci* MEAM1 colony (mtCOI GenBank accession no. GQ332577) was maintained on cotton plants (*Gossypium hirsutum* cv. Shiyuan 321) as described previously [16, 25, 50]. The *B*. *tabaci* colony harbors *Portiera*, *Hamiltonella*, and *Rickettsia* [16, 25]. The genotype of the whitefly colonies was monitored every three to five generations by Sanger sequencing of PCR-generated amplicons for the mtCOI gene. Cotton plants were grown in potting mix (Pindstrup, Denmark) supplemented with Miracle-Gro Water Soluble All Purpose Plant Food every 2–3 days. The cotton plants were grown singly in 1.5-L pots to the six-to-seven true-leaf stage for the experiments unless otherwise specified. The whitefly colony and plants were maintained in separate climate-controlled chambers, at 26 ± 2°C, 14:10 h (L:D) photoperiod and 60%-80% relative humidity (RH). LED fluorescent lights were used and light intensity in the walk-in chamber was approximately 400 μmol/m^2^sec.

### Fluorescence *in situ* hybridization (FISH)

Localization of *Portiera* and *Hamiltonella* in bacteriocytes and *Portiera* and *Rickettsia* in the ovaries of female adult whiteflies was studied by FISH using a previously described protocol [16, 25, 50, 51].

### Amino acid sequence alignment and phylogenetic tree analysis

To determine the homologous genes in other whitefly species and cultures, verified sequences of *dapB*, *dapF*, and *lysA* in *B*. *tabaci* MEAM1 were subjected to TBLASTX against the genome of *B*. *tabaci* MED [52], SSA-ECA (GenBank accession No.: GCA_004919745.1), MED-ASL, Asia I, Asia II-1, Asia II-5, Asia II-6, SSA-ECA and New World (provided by Paul Visendi and Susan Seal) and transcriptome of *B*. *tabaci* MEAM1 [9], MED [53] and Asia II 3 [54] and *Trialeurodes vaporariorum* (the National Center for Biotechnology Information (NCBI) Transcriptome Shotgun Assembly (TSA) database under the accession No.: GHMB00000000). The top TBLASTX hits were obtained. Amino acid sequence alignments for each of the three genes were conducted using BioEdit v7.1.3.0 among the ten whitefly cryptic species including *B*. *tabaci* MEAM1, MED, MED-ASL, Asia I, Asia II-1, Asia II-3, Asia II-5, Asia II-6, SSA-ECA and New World (S1 Table), *Halmiltonella* and *Rickettsia*. To construct the molecular phylogenetic tree for each of whitefly DapB, DapF, and LysA, a Bayesian inference (BI) analysis was conducted as described previously [9, 50]. Protein sequences were aligned by MAFFT 7, trimmed by trimAL v1.3 with the -automated1 flag set for likelihood-based phylogenetic methods, and manually corrected in BioEdit v7.1.3.0. Alignment lengths for dapB, dapF and LysA are 233aa, 270aa and 412 aa, respectively. The best-fit model was identified by ProtTest v2.4. The LG+I+G+F, WAG+G and LG+I+G+F model corresponding to DapB, DapF, and LysA, respectively, was used for BI analysis in MrBayes 3.2. A posterior probability of each node was used for the support value of the node. The phylogenetic trees were rooted by outgroups and graphically visualized in FigTree v1.4.0 (http://tree.bio.ed.ac.uk/software/figtree/).

### Quantitative PCR (qPCR) and qRT-PCR analysis

DNA was extracted following the Nonidet-P40-based protocol as described previously [48]. Symbionts were quantified by qPCR using the CFX96 Real-Time PCR Detection System (Bio-Rad, Hercules, USA) with 2×SYBR Green master Mix (Bio-Rad) as described previously [16, 25, 50]. *Portiera*, *Hamiltonella* and *Rickettsia* were quantified using the copy number of *16S rRNA*, *16S rRNA* and *gltA* genes, respectively, with the *B*. *tabaci β-actin* gene as the internal standard for normalization. Three technical replicates were performed for each biological replicate for symbiont elimination experiments and for gene silencing experiments. Total RNA was extracted from whitefly samples using TRI-reagents (Sigma-Aldrich, St. Louis, MO, USA) following manufacturer’s instructions. The qRT-PCR was performed as described previously [9, 16]. Relative expression was calculated using the *β-actin* gene for transcript normalization in the symbiont elimination and gene silencing experiments. Three technical replicates were performed for each biological replicate. All of the primers used in this study are shown in S2 Table. Relative symbiont density and gene expression were calculated using the 2^-ΔCt^ method [55].

### Amino acid measurement

The whole body adult whiteflies were homogenized for amino acid analysis by UPLC using the protocol described previously [25, 56]. Briefly, samples were injected into an Agilent UPLC with a PDA detector and AccQ-Tag Ultra 2.1 x 100 mm column. Amino acids are determined by comparing their retention time with standards, protein-amino acids μl^-1^ (Waters amino acid hydrolysate standard #088122, supplemented with asparagine, tryptophan, and glutamine) and quantified with standard curves. Proteins were quantified using a Lowry Protein Assay Kit (Sangon, Biotech) following manufacturer’s instructions using bovine serum albumin as a standard. Amounts of individual amino acids were normalized to the total protein content.

### Effects of *Hamiltonella* elimination by antibiotic treatment on lysine gene expression and lysine levels

To specifically eliminate *Hamiltonella*, hundreds of adult whiteflies of *B*. *tabaci* (F0, 0–7 days after emergence) were released into each feeding chamber and fed on 25% sucrose solution (w/v) supplemented with the antibiotics ampicillin, gentamycin and cefotaxime (BBI Life Sciences, Shanghai, China), each at 500 μg/mL, for four days. The artificial diets with antibiotics were renewed every two days as described previously [16, 25, 26]. Control insects were administered sucrose solution not supplemented with antibiotics. Following the antibiotic treatment, *B*. *tabaci* were transferred to cotton plants. F1 female adults at 5 d, 10 d, and 15 d after emergence were collected. The DNA was extracted from 12, 10 and 11 female adult whiteflies at 5 d, 10 d, and 15 d after emergence, respectively, and used for symbiont quantification by qPCR. The F1 *B*. *tabaci* with reduced *Hamiltonella* titers (-HBt), which were obtained by antibiotic treatment, and control F1 *B*. *tabaci* (+HBt), which were obtained by feeding sucrose solution not supplemented with antibiotics, were identified. Total RNA was extracted from 40 female adult whiteflies at day 5, 10 and 15 after emergence at the F1 stage collected from each of the four replicates and qRT-PCR was performed as described above. Relative expression of whitefly *dapB*, *dapF* and *lysA*, *Portiera dapE*, as well as *Rickettsia dapB*, *dapE* and *dapF* was calculated for whiteflies at day 5, 10 and 15 after emergence. The 25 male and 25 female adult whiteflies feeding on cotton plants at day 5, day 10 and day 15 after emergence at the F1 stage were collected for each of the six biological replicates and amino acids were extracted and quantified in the whole body of *Hamiltonella*-cured and *Hamiltonella*-infected adult whiteflies by UPLC as described above.

### Elimination of *Portiera*, *Hamiltonella* and *Rickettsia* by antibiotic treatment

To eliminate *Portiera*, *Hamiltonella* and *Rickettsia*, hundreds of adult whiteflies of *B*. *tabaci* (F0, 0–7 days after emergence) were released into each feeding chamber and fed on 25% sucrose solution (w/v) supplemented with the antibiotic rifampicin (BBI Life Sciences, Shanghai, China) dissolved in 5 mM phosphate buffer (pH 7.0), at 30 μg/mL for two days as described previously [36, 37, 50]. Control insects were administered sucrose solution not supplemented with antibiotics. Following the antibiotic treatment, *B*. *tabaci* were transferred to cotton plants. Recently emerged F1 female adults (within 1 week after emergence) were collected. DNA was extracted from eight female adult whiteflies and used for symbiont quantification by qPCR. The F1 *B*. *tabaci* with reduced titers of *Portiera*, *Hamiltonella* and *Rickettsia* (-PHRBt), which were obtained by antibiotic treatment, and control F1 *B*. *tabaci* (+PHRBt), which were obtained by feeding sucrose solution not supplemented with antibiotics, were identified.

### Recombinant enzyme generation and antibody preparation

Based on genome sequences of *B*. *tabaci* MEAM1 [29], a pair of primers including restriction enzyme sites (S2 Table) were designed to clone the open reading frame of the target gene using whitefly cDNA as the template. PCR amplified products were analyzed on 1% agarose gel, the target band was purified using a PCR purification kit (Promega, Madison, WI, USA) and the products were cloned into the pMD19-T vector (Takara, Tokyo, Japan) for verification by sequencing. Finally, the whole CDS regions of *dapB*, *dapF*, and *lysA* (Genbank accession Nos.: MT215586, MT215587, and MT215585, respectively) in our whitefly culture were obtained. The recombinant enzyme for whitefly *dapB*, *dapF*, and *lysA* was generated as described previously [16, 50]. Custom-made polyclonal antibodies against DapB (predicted size, 28 kDa), DapF (predicted size, 35 kDa) and LysA (predicted size, 48 kDa) proteins were produced by ProbeGene Life Sciences Co. Ltd. following previously described methods [13, 16, 50, 57].

### Immunofluorescence microscopy

Bacteriocytes and ovaries from female adults of +HBt and -HBt whiteflies at 7 days after emergence, bacteriocytes and ovaries from female adults of ds*GFP*, and ds*lysA*-injected whiteflies as well as guts from female adults of +PHRBt and -PHRBt whiteflies at 7 days after emergence were dissected, fixed in 4% paraformaldehyde, permeabilized with 0.2% Triton X-100 in PBS and incubated with one of the polyclonal antibodies to DapB, DapF and LysA for bacteriocytes and ovaries and LysA for guts as previously described [16, 50]. The samples were incubated with no antibodies against DapB, DapF and LysA as the negative control. Three biological replicates were conducted. Images were collected and analyzed on a FV3000 confocal microscope (Olympus, Japan).

### Functional complementation of *E*. *coli* lysine auxotrophs with whitefly HTGs

To examine the metabolic function of horizontally transferred *dapB*, *dapF*, and *lysA*, *E*. *coli* lysine gene knockout mutants were generated and functional complementation with whitefly HTGs were carried out as described previously [16, 50]. The *E*. *coli* K-12 BW25113 *dapB*, *dapF* and *lysA* knockout mutants (i.e., -Δ*dapB*, -Δ*dapF*, and -Δ*lysA*) were generated following the Lambda Red protocol as described previously [58–60]. Then *E*. *coli* wild-type K-12, mutant K-12, and mutant K-12 transformants with whitefly *dapB*, *dapF* or *lysA* were grown overnight in amino acid-deficient M9 minimal medium (Coolaber, Beijing, China) at 37°C. The cell density of all the *E*. *coli* cells were measured at OD_600_ using a microplate reader (Versa Max Molecular Devices, Silicon Valley, USA) as described previously [16, 50]. Three biological replicates were conducted.

### dsRNA preparation

The dsRNAs specific to whitefly *lysA* (ds*lysA*) and *GFP* (ds*GFP*) were synthesized using a T7 RiboMAX Express RNAi System kit (Promega, USA), following manufacturer’s instructions and purified as described previously [16, 50].

### Effects of silencing horizontally transferred *lysA* in whiteflies infected with *Hamiltonella* on lysine level, whitefly fecundity, symbiont abundance and *Hamiltonella lysA* expression

To investigate whether silencing of horizontally transferred *lysA* influences lysine levels, approximately 570 female adult whiteflies infected with *Portiera*, *Hamiltonella* and *Rickettsia* at day 4 after emergence were injected with 1.5 μg/μL ds*lysA* in injection buffer using a Eppendorf microinjection System (Hamburg, Germany) as described previously [50]. Control whiteflies were injected with ds*GFP*. The average injection volume used was 10 nl. The survival rate of injected whiteflies was 80–100% 24 h after injection. To investigate whether silencing *lysA* influences lysine level, after injection, 330 female adult whiteflies were transferred onto cotton leaf disks kept on 1.5% agar plates in the incubator at 26 ± 2°C, with 14:10 h (L:D) photoperiod and 60%-80% RH. Three biological replicates were conducted. After three days, RNA was extracted from five female adult whiteflies for each of three biological replicates to examine the expression of *lysA*. In parallel, 50 female adults of ds*GFP*, and ds*lysA*-injected whiteflies in each of three biological replicate were collected for lysine analysis as described above. The ages of whiteflies at day 3 after RNAi treatment correspond to those of other experiments in this study, and this time point was used throughout in RNAi experiments.

To investigate whether silencing *lysA* influences whitefly fecundity, approximately 70 female adult whiteflies infected with *Portiera*, *Hamiltonella* and *Rickettsia* at day 4 after emergence were injected using the microinjection procedures described above. After injection, individuals were transferred onto a leaf disk kept on the 1.5% agar plate as described above. Egg numbers were recorded for the surviving whiteflies with 22 biological replicates of individuals at day 3 post injection.

To test whether gene silencing impacts the abundance of *Portiera*, *Hamiltonella* and *Rickettsia* and *Hamiltonella lysA* expression, approximately 120 female adult whiteflies infected with *Portiera*, *Hamiltonella* and *Rickettsia* at day 4 after emergence were injected with 1.5 μg/μL ds*lysA* as described above. After injection, whiteflies were transferred onto the leaf disks kept on 1.5% agar plates as described above. After three days, DNA was extracted from individuals of ds*GFP*-treated and *lysA* RNAi whiteflies for each of 11 biological replicates and qPCR was performed as described above. RNA was extracted from 40 female adult whiteflies for each of four biological replicates to examine the expression of *Hamiltonella lysA* in RNAi whiteflies.

### Effects of silencing horizontally transferred *lysA* in whiteflies lacking *Hamiltonella* on lysine levels, whitefly fecundity and symbiont abundance

To detect effects of silencing horizontally transferred *lysA* in whiteflies lacking *Hamiltonella* on lysine levels, whitefly fecundity and symbiont abundance, *Hamiltonella* was specifically eliminated as described above. The DNA was extracted from ten female adult whiteflies, and used for symbiont quantification by qPCR. To investigate whether silencing of horizontally transferred *lysA* influences lysine level, after whiteflies lacking *Hamiltonella* within 6 days after emergence were injected with 1.5 μg/μL ds*lysA*, individuals were transferred onto a cotton leaf disk kept on the 1.5% agar plate as described above. After three days, RNA was extracted from five female adult whiteflies for each of three biological replicates to examine the expression of *lysA*. 50 female adults of ds*GFP* and ds*lysA*-injected -HBt whiteflies in each of four biological replicate were collected for lysine analysis as described above. To investigate whether silencing *lysA* in whiteflies lacking *Hamiltonella* influences whitefly fecundity, approximately 150 female adult whiteflies lacking *Hamiltonella* within 6 days after emergence were injected using the microinjection procedures described above. After injection, individuals were transferred onto a leaf disk kept on the 1.5% agar plate as described above, and allowed to lay eggs for three days. Egg numbers were recorded with 14 biological replicates of individuals.

To test whether gene silencing impacts the abundance of *Portiera* and *Rickettsia* in whitefly ovarioles, approximately 100 female adult whiteflies lacking *Hamiltonella* within 6 days after emergence were injected with 1.5 μg/μL ds*lysA* as described above. After injection, whiteflies were transferred onto the cotton leaf disks kept on 1.5% agar plates as described above. After three days, whiteflies were collected and ovarioles were dissected. DNA was extracted from individual ovarioles of ds*GFP*-treated and *lysA* RNAi whiteflies for each of 11 biological replicates and qPCR was performed as described above.

### Statistical analyses

The OD values of the *E*. *coli* wild-type K-12, mutant K-12 and mutant K-12 transformants were compared using one-way ANOVA at a significance threshold of 0.05 followed by LSD post-hoc tests. For symbiont titer, gene expression level, lysine amount as well as the egg numbers of ds*GFP*, and ds*lysA*-injected female whiteflies, statistical differences were evaluated using one-way ANOVA at a significance threshold of 0.05. Percentage data were arcsine square root transformed before analysis. All of the data analyses were conducted using the STATISTICA v6.1 software (StatSoft, Inc., Tulsa, OK, USA).

## Supporting information

S1 Fig Amino acid sequence alignment between long and short LysA protein in *B*. *tabaci* MEAM1 and conserved domains of two proteins. (A) Amino acid sequence alignment between long (Bta03593) and short (Bta03589) LysA protein in *B*. *tabaci* MEAM1. (B,C) Conserved domains of long (B) and short (C) LysA protein. The conserved domain was presented based on the result of BLASTP.
(TIF)

S2 Fig Amino acid sequence alignment of DapB acquired horizontally in *B*. *tabaci* MEAM1, MED, MED-ASL, Asia I, Asia II-1, Asia II-3, Asia II-5, Asia II-6, New World and SSA-ECA. (TIF)

S3 Fig Amino acid sequence alignment of DapF acquired horizontally in *B*. *tabaci* MEAM1, MED, MED-ASL, Asia I, Asia II-1, Asia II-3, Asia II-5, Asia II-6, New World and SSA-ECA. (TIF)

S4 Fig Amino acid sequence alignment of LysA acquired horizontally in *B*. *tabaci* MEAM1, MED, MED-ASL, Asia I, Asia II-1, Asia II-3, Asia II-5, Asia II-6, New World and SSA-ECA. (TIF)

S5 Fig Phylogenetic tree analysis of horizontally transferred DapB in whiteflies. Posterior probabilities estimated using Bayesian inference methods are shown at each node. Collapsed branches are shown as triangular wedges with the number of sequences shown inside the wedge. The scale bar reflects evolutionary distance, measured in units of substitution per amino acid site.
(TIF)

S6 Fig Phylogenetic tree analysis of horizontally transferred DapF in whiteflies. Posterior probabilities estimated using Bayesian inference methods are shown at each node. Collapsed branches are shown as triangular wedges with the number of sequences shown inside the wedge. The scale bar reflects evolutionary distance, measured in units of substitution per amino acid site.
(TIF)

S7 Fig Phylogenetic tree analysis of horizontally transferred LysA in whiteflies. Posterior probabilities estimated using Bayesian inference methods are shown at each node. Collapsed branches are shown as triangular wedges with the number of sequences shown inside the wedge. The scale bar reflects evolutionary distance, measured in units of substitution per amino acid site.
(TIF)

S8 Fig SDS–PAGE and western blot analysis. (A-C) SDS-PAGE electrophoretic separation of fractions after affinity chromatography for purified recombinant protein of whitefly DapB (A), DapF (B), and LysA (C). M represents molecular mass standards. Lane 0–5 represents cell pellet, supernatant of lysis buffer, flow-through, wash-unbound, eluted protein and residue, respectively. (D-F) The specificity of polyclonal antibodies verified by western blot using anti-DapB antibody (D), anti-DapF antibody (E), and anti-LysA antibody (F). Lane 1–3 represents 1 ng, 2 ng and 5 ng of purified recombinant protein loaded in SDS-PAGE, respectively.
(TIF)

S9 Fig Localization of DapB, DapF, and LysA (green) in bacteriocytes (A-C) and ovaries (D-F) of female adult whiteflies. +HBt and -HBt represent *Hamiltonella*-infected and *Hamiltonella*-cured whiteflies, respectively. n = 3. The samples were incubated with no antibodies against DapB, DapF, and LysA as the negative control. DNA was stained with DAPI.
(TIF)

S10 Fig Effects of *Portiera*, *Hamiltonella* and *Rickettsia* elimination on LysA localization in guts of *B*. *tabaci*. (A) Effects of antibiotic treatments on the abundance of symbionts in *B*. *tabaci*. n = 8. The significant differences between treatments are indicated by asterisks (**P < 0.01; ***P < 0.001). (B,C) Localization of LysA proteins in guts of female adult whiteflies of +PHRBt and -PHRBt. n = 3. DNA was stained with DAPI. +PHRBt and -PHRBt represent *Portiera*, *Hamiltonella* and *Rickettsia*-infected and *Portiera*, *Hamiltonella* and *Rickettsia*-cured whiteflies, respectively.
(TIF)

S11 Fig Expression of whitefly *lysA* at day 3 after *Hamiltonella*-infected (A) (+HBt) and *Hamiltonella*-cured (B) (-HBt) whiteflies were microinjected with ds*lysA*. n = 3. The significant differences between treatments are indicated by asterisks (**P < 0.01; ***P < 0.001).
(TIF)

S1 Table Horizontally transferred lysine genes in the whitefly *B*. *tabaci* and *T. vaporarium*. (DOCX)

S2 Table Primers used in this study. (DOCX)

S1 Data Genes involved in essential amino acid synthesis in *Rickettsia* of the whitefly *B*. *tabaci* MEAM1. (XLSX)

S2 Data Genes involved in lysine synthesis in *Portiera* and *Hamiltonella* of the whitefly *B*. *tabaci* MEAM1. (XLSX)
