# Supplementary figures and images for: Lysine provisioning by horizontally acquired genes promotes mutual dependence between whitefly and two intracellular symbionts
Source: PLoS Pathog. 2021 Nov 29;17(11):e1010120. doi: 10.1371/journal.ppat.1010120 (PMC8659303; doi:10.1371/journal.ppat.1010120)

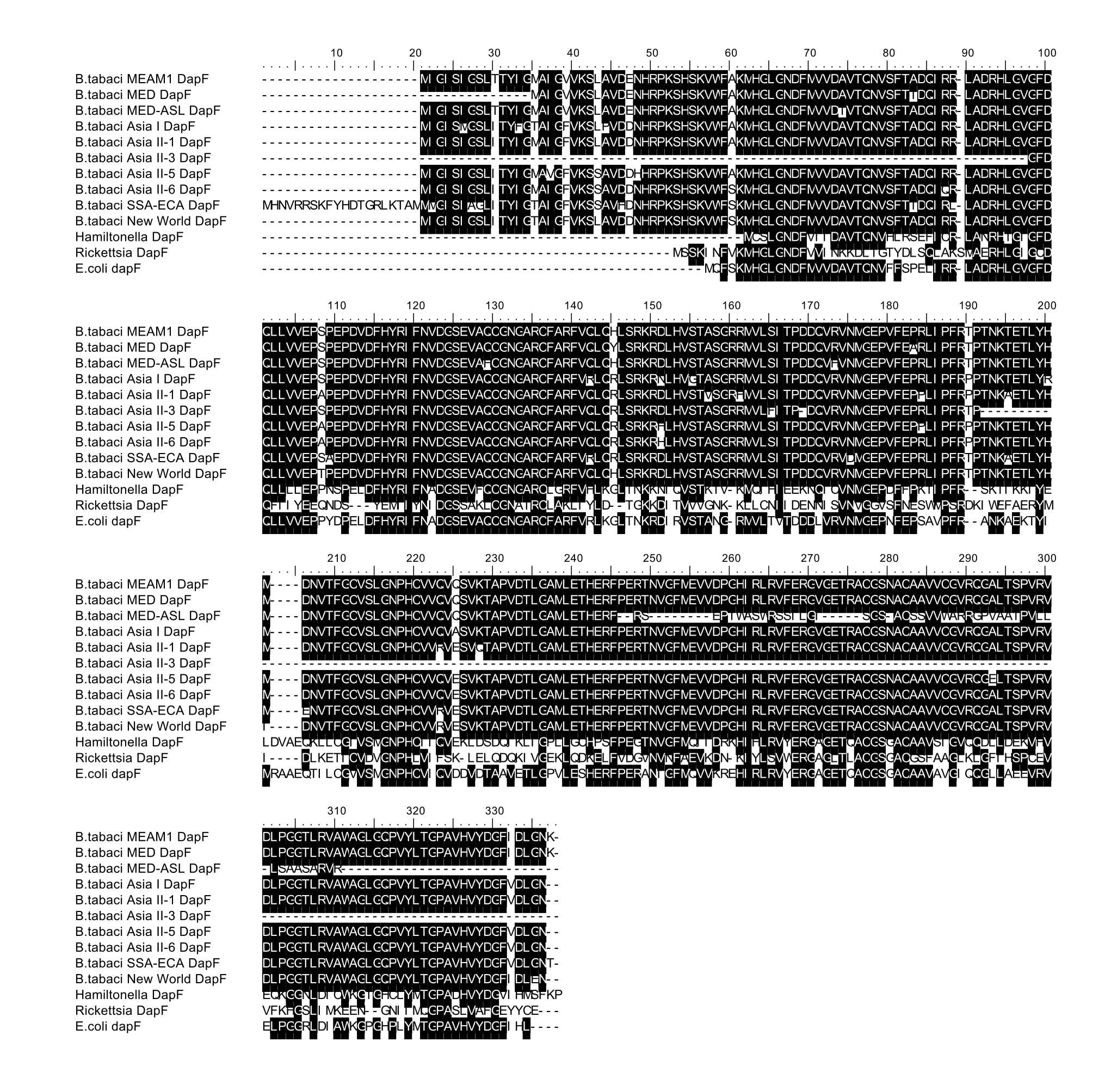

Supplement: S3 Fig — (TIF) [file ppat.1010120.s003.tif]

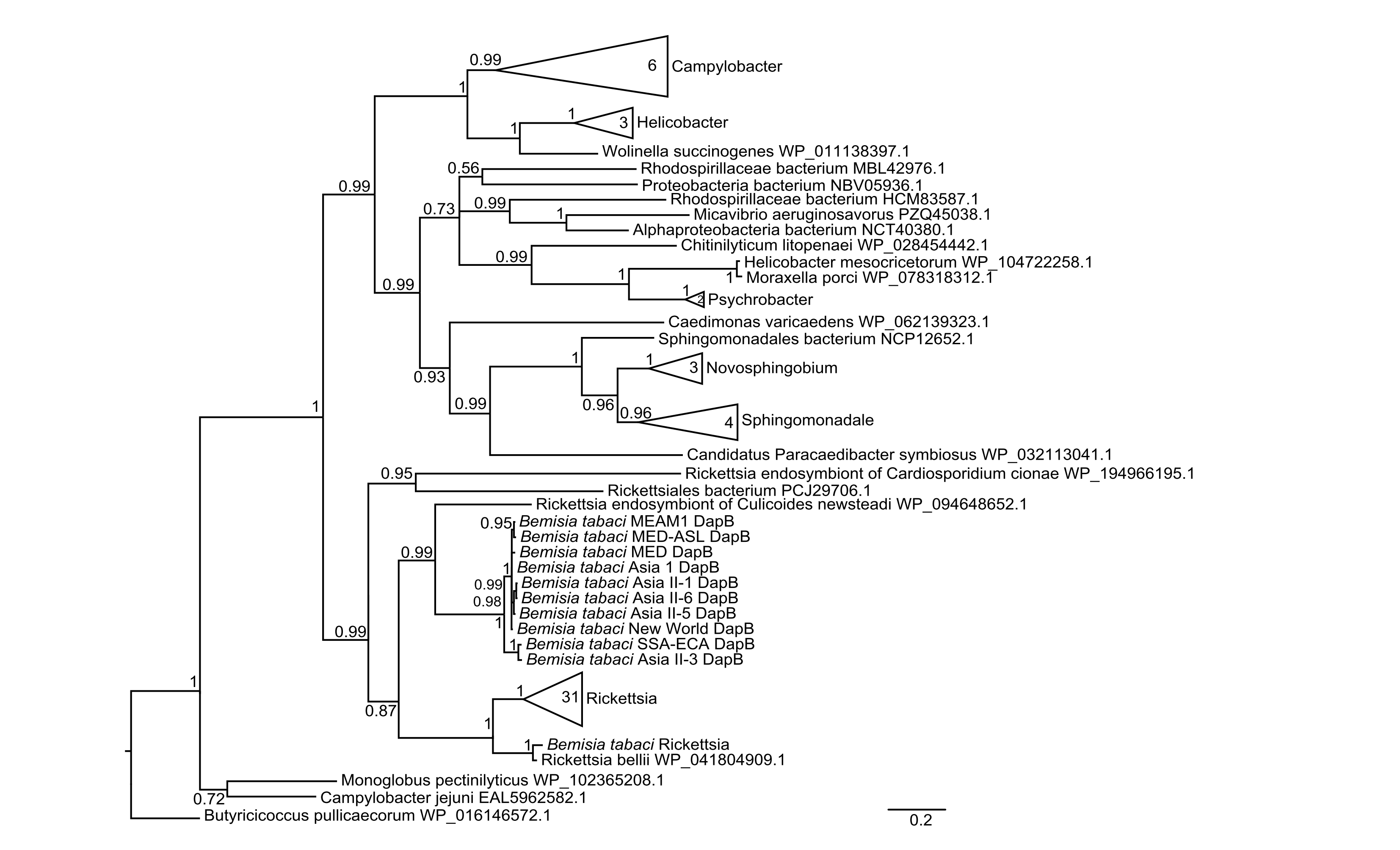

Supplement: S5 Fig — Posterior probabilities estimated using Bayesian inference methods are shown at each node. Collapsed branches are shown as triangular wedges with the number of sequences shown inside the wedge. The scale bar reflects evolutionary distance, measured in units of substitution per amino acid site. (TIF) [file ppat.1010120.s005.tif]

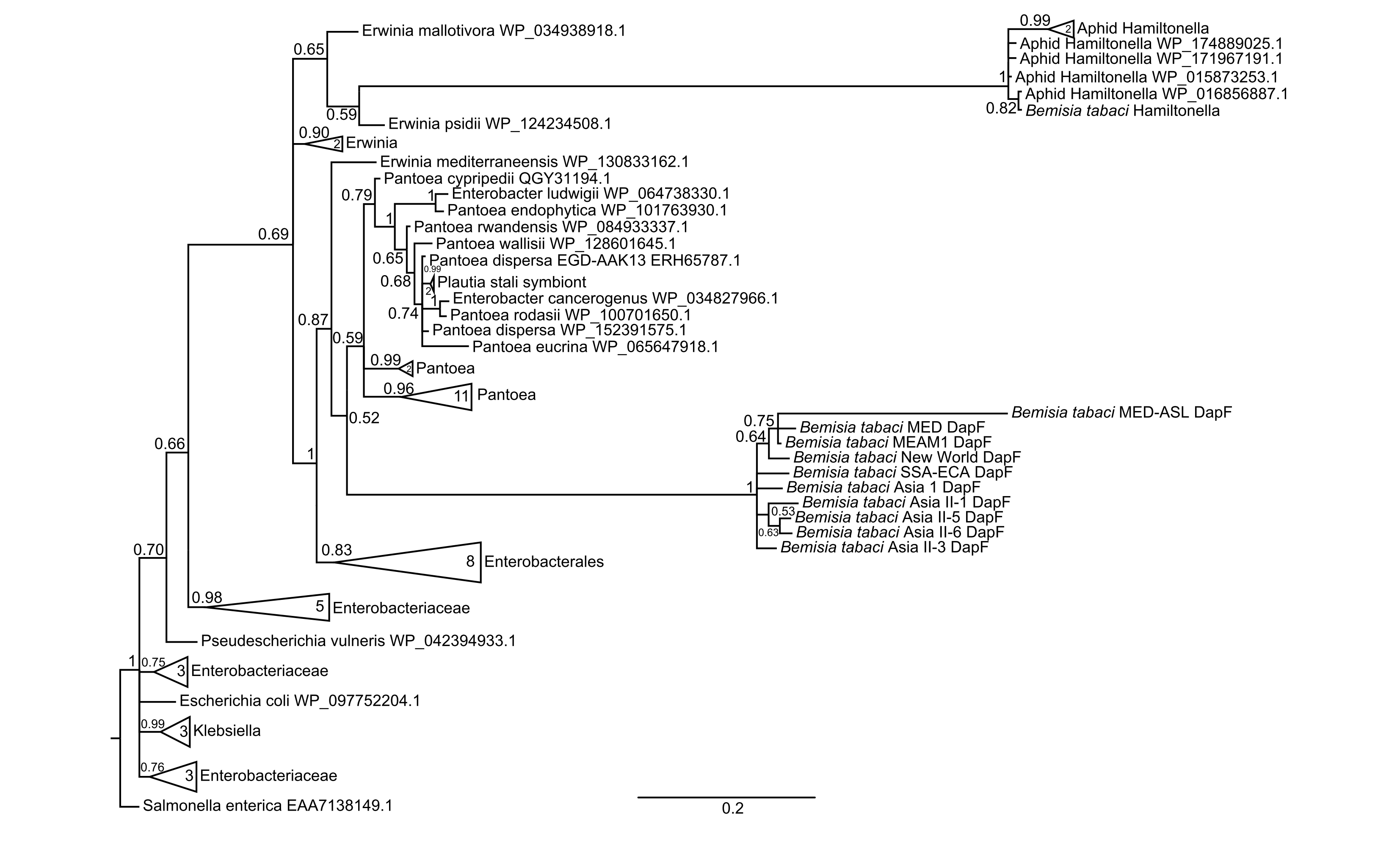

Supplement: S6 Fig — Posterior probabilities estimated using Bayesian inference methods are shown at each node. Collapsed branches are shown as triangular wedges with the number of sequences shown inside the wedge. The scale bar reflects evolutionary distance, measured in units of substitution per amino acid site. (TIF) [file ppat.1010120.s006.tif]

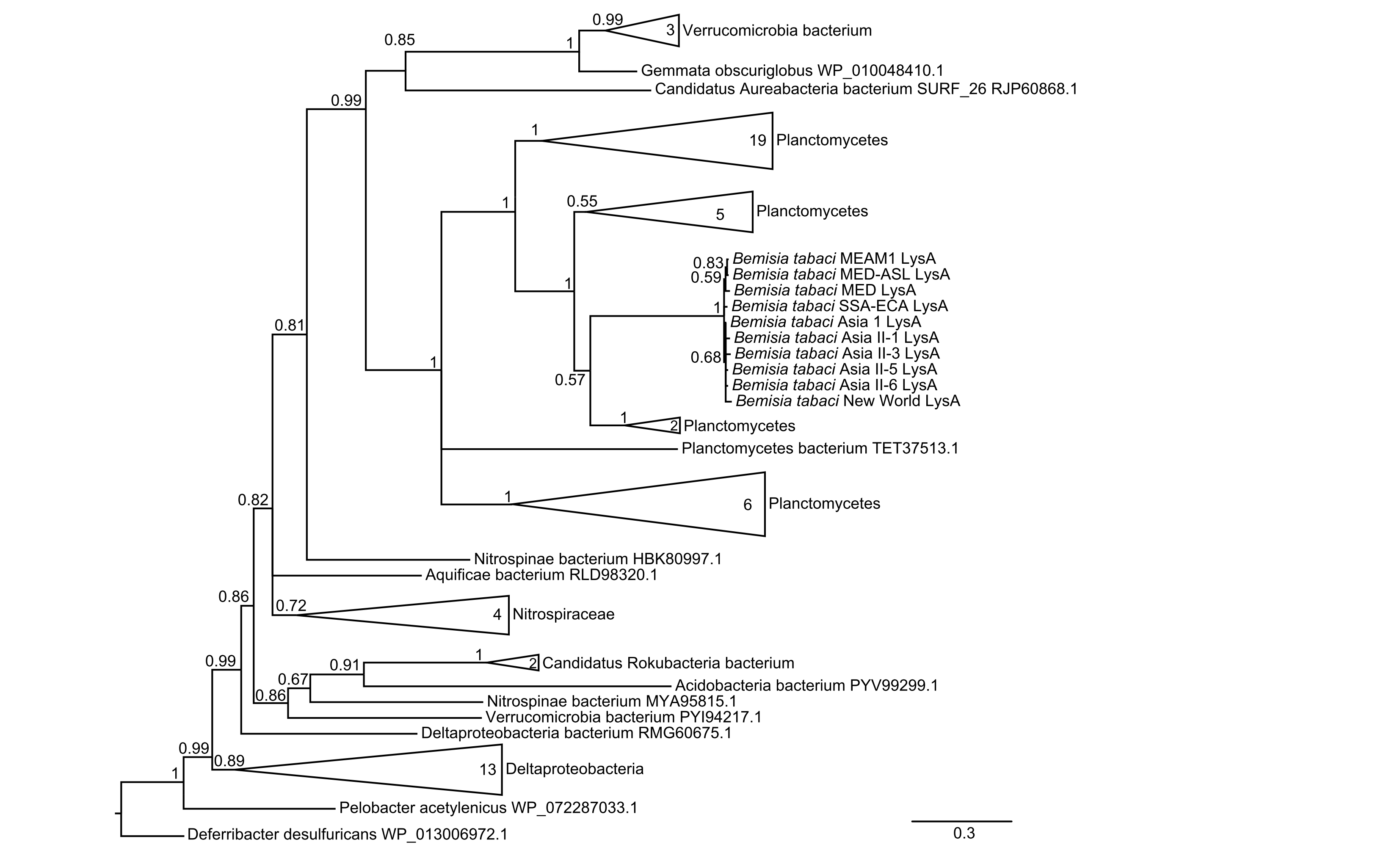

Supplement: S7 Fig — Posterior probabilities estimated using Bayesian inference methods are shown at each node. Collapsed branches are shown as triangular wedges with the number of sequences shown inside the wedge. The scale bar reflects evolutionary distance, measured in units of substitution per amino acid site. (TIF) [file ppat.1010120.s007.tif]

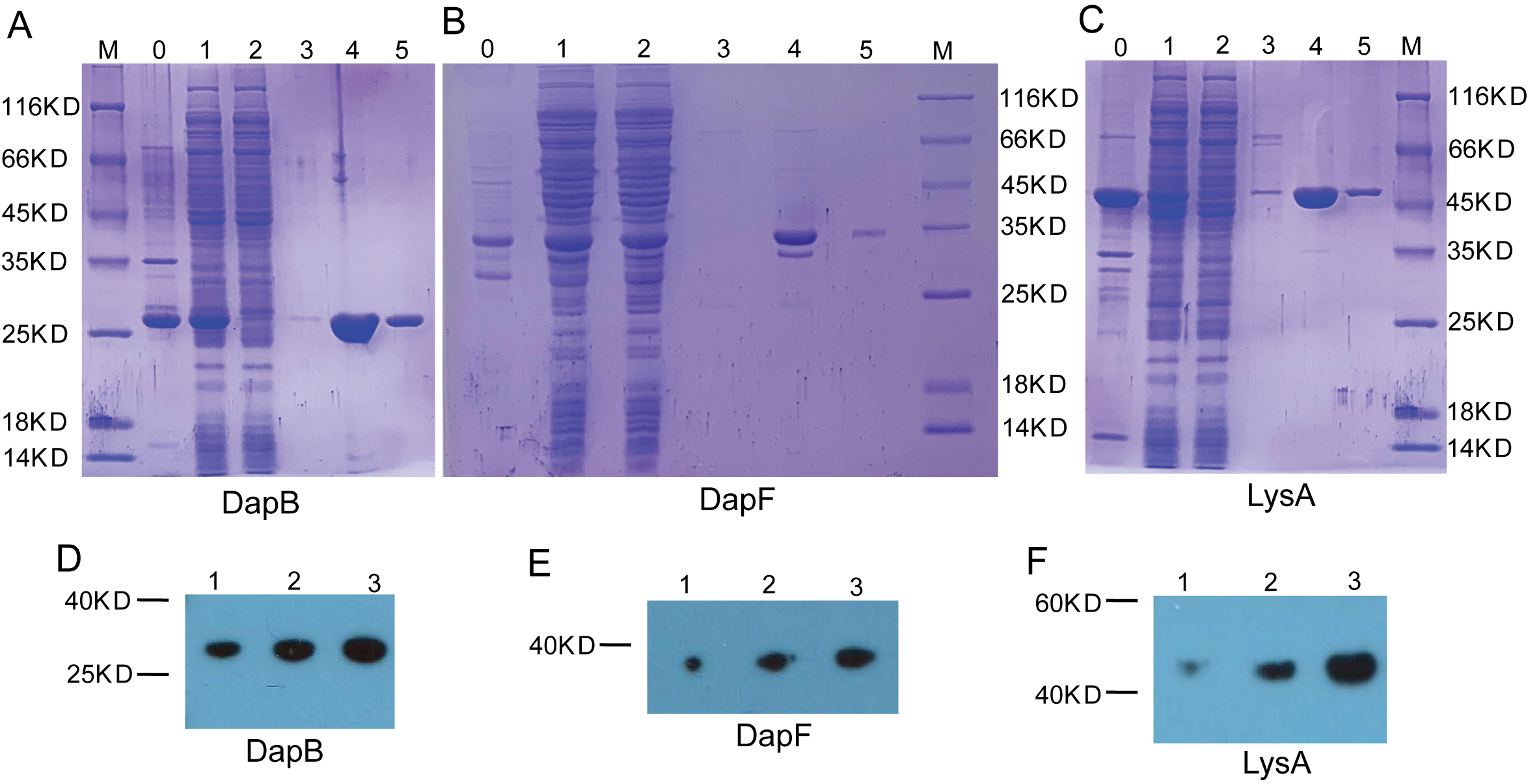

Supplement: S8 Fig — (A-C) SDS-PAGE electrophoretic separation of fractions after affinity chromatography for purified recombinant protein of whitefly DapB (A), DapF (B), and LysA (C). M represents molecular mass standards. Lane 0–5 represents cell pellet, supernatant of lysis buffer, flow-through, wash-unbound, eluted protein and residue, respectively. (D-F) The specificity of polyclonal antibodies verified by western blot using anti-DapB antibody (D), anti-DapF antibody (E), and anti-LysA antibody (F). Lane 1–3 represents 1 ng, 2 ng and 5 ng of purified recombinant protein loaded in SDS-PAGE, respectively. (TIF) [file ppat.1010120.s008.tif]

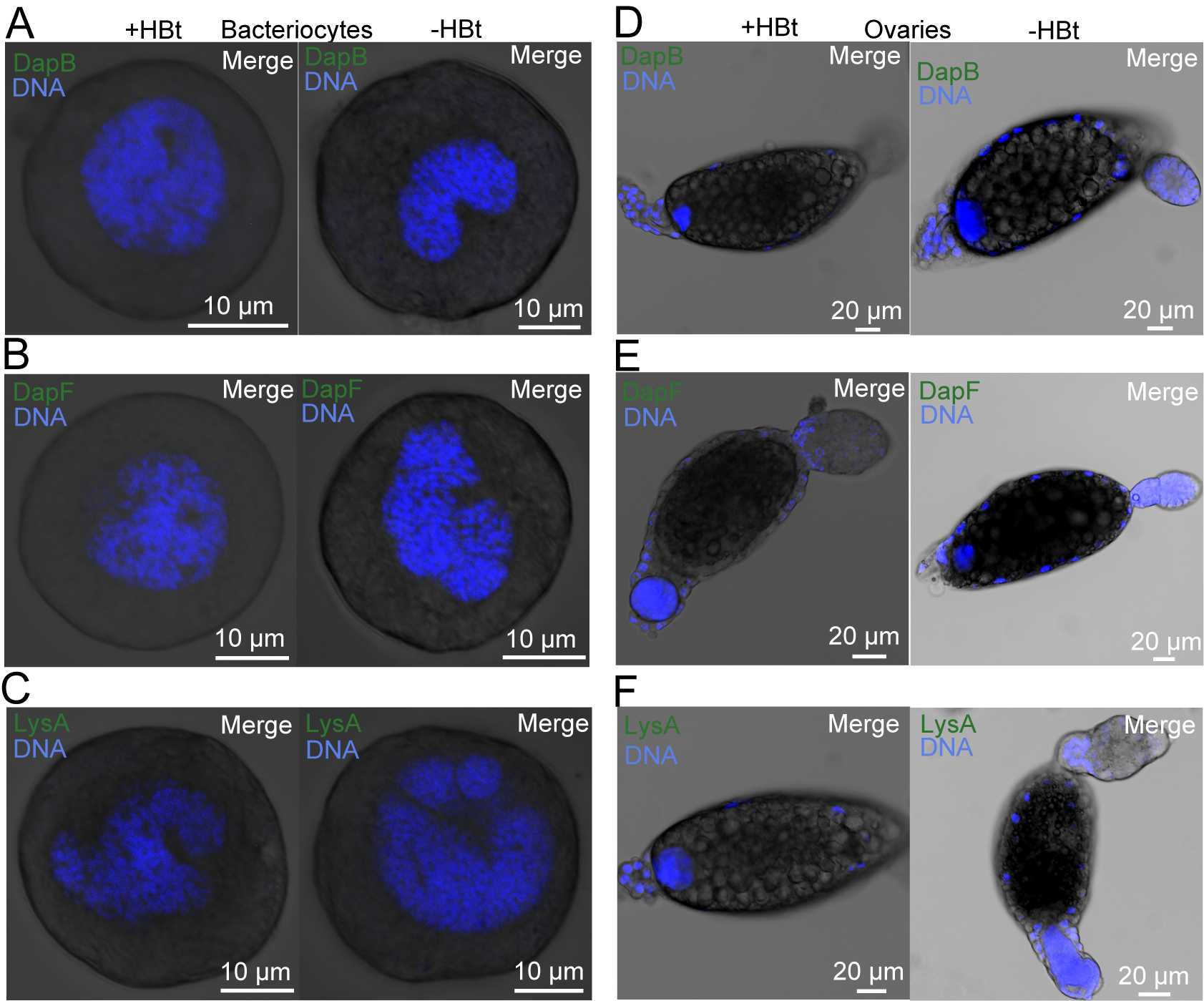

Supplement: S9 Fig — +HBt and -HBt represent Hamiltonella-infected and Hamiltonella-cured whiteflies, respectively. n = 3. The samples were incubated with no antibodies against DapB, DapF, and LysA as the negative control. DNA was stained with DAPI. (TIF) [file ppat.1010120.s009.tif]

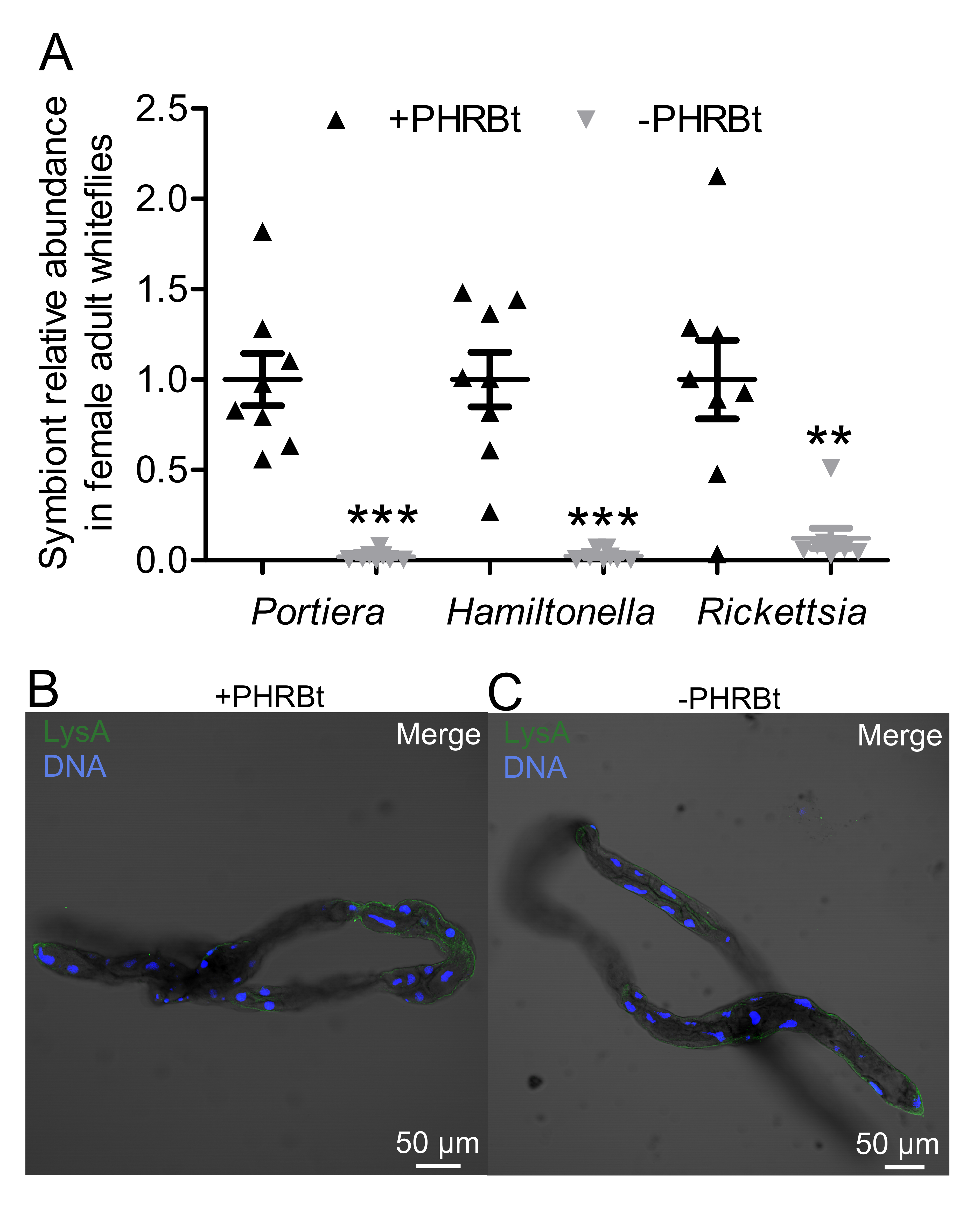

Supplement: S10 Fig — (A) Effects of antibiotic treatments on the abundance of symbionts in B. tabaci. n = 8. The significant differences between treatments are indicated by asterisks (**P < 0.01; ***P < 0.001). (B,C) Localization of LysA proteins in guts of female adult whiteflies of +PHRBt and -PHRBt. n = 3. DNA was stained with DAPI. +PHRBt and -PHRBt represent Portiera, Hamiltonella and Rickettsia-infected and Portiera, Hamiltonella and Rickettsia-cured whiteflies, respectively. (TIF) [file ppat.1010120.s010.tif]

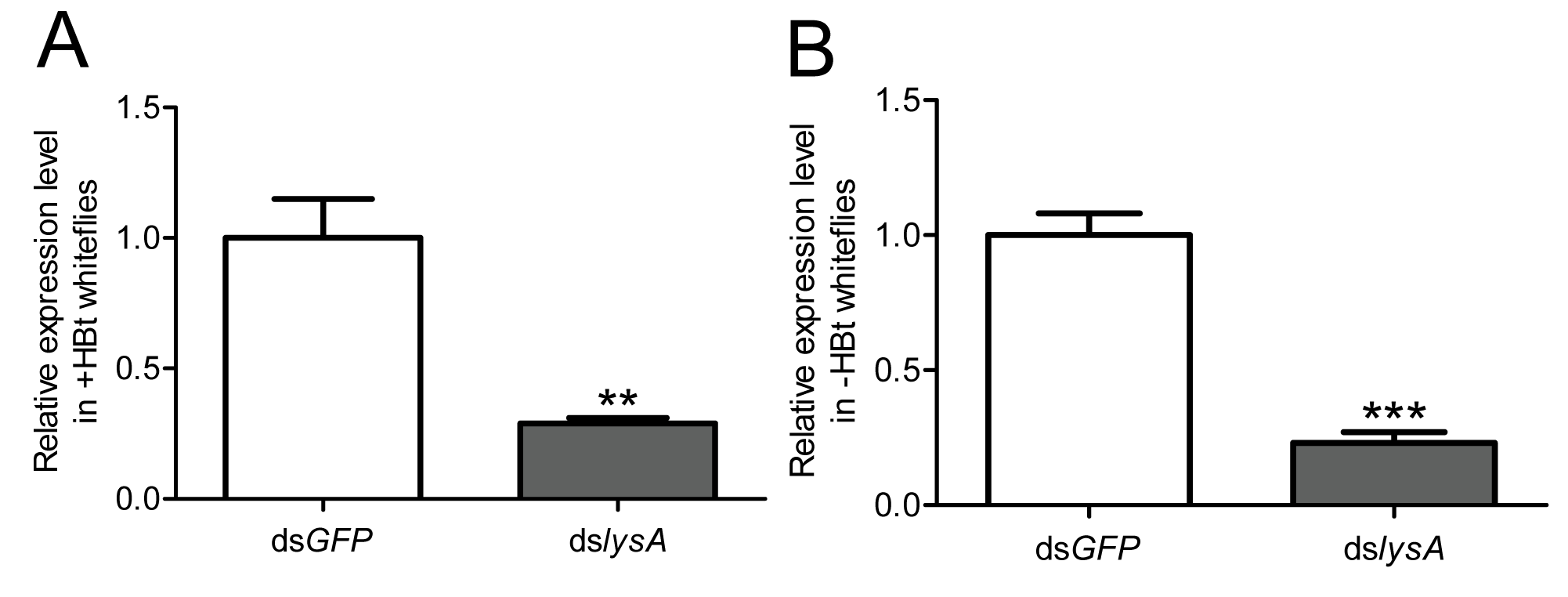

Supplement: S11 Fig — n = 3. The significant differences between treatments are indicated by asterisks (**P < 0.01; ***P < 0.001). (TIF) [file ppat.1010120.s011.tif]
