# Supplementary material for: Lysine provisioning by horizontally acquired genes promotes mutual dependence between whitefly and two intracellular symbionts
Source: PLoS Pathog. 2021 Nov 29;17(11):e1010120. doi: 10.1371/journal.ppat.1010120 (PMC8659303; doi:10.1371/journal.ppat.1010120)
Supplement: S1 Table — (DOCX) [file ppat.1010120.s012.docx]

| **S1 Table** Horizontally transferred lysine genes in the whitefly *B. tabaci* and *Trialeurodes vaporarium* | | | | |
| --- | --- | --- | --- | --- |
| **Species** | **Gene name** | **Gene ID** | **Gene ID** | **Gene ID** |
| *B. tabaci* MEAM1 | *dapB* | [Bta20020](http://www.whiteflygenomics.org/cgi-bin/bta/geneinfo.cgi?&gene=Bta20020)[1] | CL592.Contig2_AB[2] | MT215586[3] |
|  | *dapF* | [Bta06657](http://www.whiteflygenomics.org/cgi-bin/bta/geneinfo.cgi?&gene=Bta06657)[1] | Unigene15642_AB[2] | MT215587[3] |
|  | *lysA* | [Bta03593](http://www.whiteflygenomics.org/cgi-bin/bta/geneinfo.cgi?&gene=Bta03593)[1] | CL4744.Contig1_AB[2] | MT215585[3] |
| *B. tabaci* MED | *dapB* | Genomic scaffold_733[4] | Singletons21168[5] |  |
|  | *dapF* | Genomic scaffold_265[4] | Singletons11119[5] |  |
|  | *lysA* | Genomic scaffold_691[4] | Singletons4610[5] |  |
| *B. tabaci* MED-ASL | *dapB* | MW573859 |  |  |
|  | *dapF* | MW573847 |  |  |
|  | *lysA* | MW573851 |  |  |
| *B. tabaci* Asia I | *dapB* | MW573856 |  |  |
|  | *dapF* | MW573843 |  |  |
|  | *lysA* | MW573850 |  |  |
| *B. tabaci* Asia II-1 | *dapB* | MW573855 |  |  |
|  | *dapF* | MW573844 |  |  |
|  | *lysA* | MW573849 |  |  |
| *B. tabaci* Asia II-3 | *dapB* | BT_ZHJ1_ZJU_Unigene20879[6] |  |  |
|  | *dapF* | BT_ZHJ1_ZJU_Unigene27765[6] |  |  |
|  | *lysA* | BT_ZHJ1_ZJU_Unigene223[6] |  |  |
| *B. tabaci* Asia II-5 | *dapB* | MW573857 |  |  |
|  | *dapF* | MW573845 |  |  |
|  | *lysA* | MW573852 |  |  |
| *B. tabaci* Asia II-6 | *dapB* | MW573858 |  |  |
|  | *dapF* | MW573846 |  |  |
|  | *lysA* | MW573853 |  |  |
| *B. tabaci* SSA-ECA | *dapB* | Genomic [scaffold_570](http://www.whiteflygenomics.org/cgi-bin/bta/geneinfo.cgi?type=gene&gene=scaffold570)[7] |  |  |
|  | *dapF* | Genomic [scaffold_11675](http://www.whiteflygenomics.org/cgi-bin/bta/geneinfo.cgi?type=gene&gene=Ssa11675)[7] |  |  |
|  | *lysA* | Genomic scaffold[_05826](http://www.whiteflygenomics.org/cgi-bin/bta/geneinfo.cgi?type=gene&gene=Ssa05826)[7] |  |  |
| *B. tabaci* New World | *dapB* | MW573854 |  |  |
|  | *dapF* | MW573842 |  |  |
|  | *lysA* | MW573848 |  |  |
| *T. vaporariorum* | *dapB* | Absent[8] | Absent[9] |  |
|  | *dapF* | Absent[8] | Absent[9] |  |
|  | *lysA* | Absent[8] | Absent[9] |  |
| Note: the whitefly *B. tabaci* and *Trialeurodes vaporarium* are phylogenetically-distant whitefly species[10].  **Notes:**  **1.** The sequences are from the genome of the whitefly *B. tabaci* MEAM1 culture collected in USA (Chen et al. 2016 BMC Biology 14:110 )  **2.** The sequences are from the bacteriocyte transcriptome of the whitefly *B. tabaci* MEAM1 culture (mtCO1 GenBank accession no.GQ332577) collected in China (Luan et al. 2015 GBE 7:2635-2647)  **3.** The sequences are verified from the whole body of the whitefly *B. tabaci* MEAM1 culture (mtCO1 GenBank accession no.GQ332577) collected in China  **4.** The sequences are from the genome of the whitefly *B. tabaci* Mediterranean culture collected in China (Xie et al. 2017 Gigascience 6,1-7)  **5.** The sequences are from the transcriptome of the whitefly *B. tabaci* Mediterranean culture collected in China (Wang et al. 2010 BMC Genomics 11,400)  **6.** The sequences are from the transcriptome of the whitefly *B. tabaci* Asia II 3 culture collected in China (Wang et al. 2012 BMC Genomics 13,529)  **7.** The sequences are from the genome of the whitefly *B. tabaci* SSA1 culture collected in Africa (GenBank assembly accession no.: GCA_004919745.1)  **8.** The sequences are absent from the transcriptome of the greenhouse whitefly, *T. vaporariorum* culture collected in Shenyang, China (NCBI Transcriptome Shotgun Assembly database (TSA) database under the accession number of GHMB00000000)  **9.** The sequences are absent from the transcriptome of the greenhouse whitefly, *T. vaporariorum* culture collected from Turkey (Karatolos et al. 2011 BMC Genomics 12,56)  **10.** Baumann P. Biology of bacteriocyte-associated endosymbionts of plant sap-sucking insects. Annu Rev Microbiol. 2005; 59:155-89. | | | | |
