# Supplementary material for: Lysine provisioning by horizontally acquired genes promotes mutual dependence between whitefly and two intracellular symbionts
Source: PLoS Pathog. 2021 Nov 29;17(11):e1010120. doi: 10.1371/journal.ppat.1010120 (PMC8659303; doi:10.1371/journal.ppat.1010120)
Supplement: S2 Table — (DOCX) [file ppat.1010120.s013.docx]

**S2 Table** Primers used in this study

| Gene name | Application | | Forward (5'-3') | | | Reverse (5'-3') | | Ampliﬁcation efficiency (%) |
| --- | --- | --- | --- | --- | --- | --- | --- | --- |
| 1. qRT-PCR | | | | | | | | |
| qRT-Bt-*dapB* | Whitefly gene expression | TGCTCAAATCAGCCATAA | | AGCAGTGCCAGAAGGAA | | | 98.62 | |
| qRT-Bt-*dapF* | Whitefly gene expression | TTCACGGCGGACCAGAT | | TGTGCGGAAGGGTATGAGT | | | 98.43 | |
| qRT-Bt-*lysA* | Whitefly gene expression | GAAGCGTGTCAGTGTCG | | TGAATATCCATGCCAAAC | | | 102.38 | |
| qRT-P-*dapE* | *Portiera* gene expression | ACATTGTTATTTGTTGGTCATACAG | | TCATATCGACAATTCCACGTGAT | | | 84.45 | |
| qRT-R-*dapB* | *Rickettsia* gene expression | AGAAGCTCATCATCGGGCTA | | CGCCACGAAGCGAAGAAATA | | | 125.25 | |
| qRT-R-*dapE* | *Rickettsia* gene expression | AATAGCGCTGCCGATAGTTT | | GTGCAACGTCTGATAATAAACCG | | | 85.79 | |
| qRT-R-*dapF* | *Rickettsia* gene expression | GTGGAGCAGGGCTAACTCTT | | AAACCAGCAGCAAAGCTACC | | | 87.42 | |
| qRT-H-*lysA* | *Hamiltonella* gene expression | TTTACGGCCGATCTCTTGGA | | TCCATGACCAAAGCCAGGAT | | | 106.17 | |
| qRT-actin[1] | Reference gene | TGGAGATGGTGTTTCCCACAC | | CCAGCCAAGTCCAAACGAAG | | |  | |
| 1. qPCR | |  | |  | | |  | |
| H-16S[2] | *Hamiltonella* density | GCATCGAGTGAGCACAGTTT | | TATCCTCTCAGACCCGCTAGA | | |  | |
| Port73/Port266[3] | *Portiera* density | GTGGGGAATAACGTACGG | | CTCAGTCCCAGTGTGGCTG | | |  | |
| glt375[3] | *Rickettsia* density | TGGTATTGCATCGCTTTGGG | | TTTCTTTAAGCACTGCAGCACG | | |  | |
| q-actin[4] | Reference gene | TCTTCCAGCCATCCTTCTTG | | CGGTGATTTCCTTCTGCATT | | |  | |
| 1. dsRNA synthesis | |  | |  | | |  | |
| ds*lysA* | Gene silencing | GGATCCTAATACGACTCACTATAGGGATGGCCGGAATAGTGC | | GGATCCTAATACGACTCACTATAGGGTGCACTGCTGTAAGGGGC | | |  | |
| ds*GFP* | Gene silencing | GGATCCTAATACGACTCACTATAGGGCACAAGTTCAGCGTGTCCG | | GGATCCTAATACGACTCACTATAGGGGTTCACCTTGATGCCGTTC | | |  | |
| 1. Recombinant protein | |  | |  | | |  | |
| RP*dapB* | Recombinant protein | CATGCCATGGGCATAAATATTGGATTATGTGGTTCTA | | CATGCTCGAGACCGAGTTGACTAAGTACATCACGCAT | | |  | |
| RP*dapF* | Recombinant protein | CATGCCATGGGCATCGGAATCTCCATAGGTAGCCT | | CATGCTCGAGTTTATTTCCTAAATCAATGAAGCCATCA | | |  | |
| RP*lysA* | Recombinant protein | CATGCCATGGCCGGAATAGTGCCACTTTCGAATCAC | | CATGCTCGAGACTAAGAGGATTTAGCTCCTCGGC | | |  | |
| 1. Complementation study | | | | | | | | |
| GK[5] | Amplification of kanamycin resistance site of E. co*li* G11 | ATTCCGGGGATCCGTCGACC | | | TGTAGGCTGGAGCTGCTTCG | |  | |
| M-*dapB* | *dapB* knockout | TCTGAAAACGGTCTATGCAAATTAACAAAAGAGAATAGCTATTCCGGGGATCCGTCGACC | | | TTAAATGTGTTATTTTTGCACCATAACAAATATTTTGTGGTGTAGGCTGGAGCTGCTTCG | |  | |
| A-*dapB* | Amplification of *dapB* from *E. coli* K12 | ATGCATGATGCAAACATCCGCG | | | TTACAAATTATTGAGATCAAGTAC | |  | |
| V-*dapB* | Verification of mutant | ATGTTATTGGCATGCAGTC | | | TGTAGGCTGGAGCTGCTTCG | |  | |
| M-*dapF* | *dapF* knockout | CTAAAAGTCAGTTTCTGTACCCGCGTGATTGGAGTAAATGATTCCGGGGATCCGTCGACC | | | AGCTCCGTGAGTGTTTCCTGCAGTTCTTCCCCTGGTTGCTTGTAGGCTGGAGCTGCTTCG | |  | |
| A-*dapF* | Amplification of *dapF* from *E. coli* K12 | ATGCAGTTCTCGAAAATGCATGG | | | TCATAGATGAATAAATCCGTCGTAG | |  | |
| V-*dapF* | Verification of mutant | ATGGTCCATCCCAGGTGAAT | | | TGTAGGCTGGAGCTGCTTCG | |  | |
| M-*lysA* | *lysA* knockout | GCTGGAGGCAAGTCATCATGCAACCAGCGACTAACCGCAGATTCCGGGGATCCGTCGACC | | | TGTGGCGTAATCATAAAAAAGCACTTATCTGGAGTTTGTTTGTAGGCTGGAGCTGCTTCG | |  | |
| A-*lysA* | Amplification of *lysA* from *E. coli* K12 | TTAAAGCAATTCCAGCGCCAGT | | | ATGCCACATTCACTGTTCAGC | |  | |
| V-*lysA* | Verification of mutant | GTCTTTAGTCCGACGCTGGTAC | | | TGTAGGCTGGAGCTGCTTCG | |  | |

**References:**

1. Wang ZZ, Shi M, Ye XQ, Chen MY, Chen XX. Identification, characterization and expression of a defensin-like antifungal peptide from the whitefly *Bemisia tabaci* (gennadius) (hemiptera: aleyrodidae). Insect Mol Biol. 2013; 22(3):297-305.
2. Brumin M, Kontsedalov S, Ghanim M. *Rickettsia* influences thermotolerance in the whitefly *Bemisia tabaci* B biotype. Insect Sci. 2011; 18, 57-66.
3. Caspi-Fluger A, Inbar M, Mozes-Daube N, Mouton L, Hunter MS, Zchori-Fein E. *Rickettsia* ‘in’ and ‘out’: two different localization patterns of a bacterial symbiont in the same insect species. PLoS One. 2011; 6(6):e21096.
4. Sinisterra XH, McKenzie CL, Hunter WB, Powell CA, Shatters RG. Differential transcriptional activity of plant-pathogenic begomoviruses in their whitefly vector (*Bemisia tabaci*, Gennadius: Hemiptera Aleyrodidae). J Gen Virol. 2005; 86(Pt 5):1525-1532.
5. Mori H, Baba T, Yokoyama K, Takeuchi R, Nomura W, Makishi K, et al. Identification of essential genes and synthetic lethal gene combinations in *Escherichia coli* k-12. Methods Mol Biol. 2015; 1279:45-65**.**
